# Prediction of Protein-Protein Interactions Related to Protein Complexes Based on Protein Interaction Networks

**DOI:** 10.1155/2015/259157

**Published:** 2015-02-03

**Authors:** Peng Liu, Lei Yang, Daming Shi, Xianglong Tang

**Affiliations:** ^1^School of Computer Science and Technology, Harbin Institute of Technology, Harbin 150001, China; ^2^Information and Network Administration Center, Heilongjiang University, Harbin 150080, China

## Abstract

A method for predicting protein-protein interactions based on detected protein complexes is proposed to repair deficient interactions derived from high-throughput biological experiments. Protein complexes are pruned and decomposed into small parts based on the adaptive *k*-cores method to predict protein-protein interactions associated with the complexes. The proposed method is adaptive to protein complexes with different structure, number, and size of nodes in a protein-protein interaction network. Based on different complex sets detected by various algorithms, we can obtain different prediction sets of protein-protein interactions. The reliability of the predicted interaction sets is proved by using estimations with statistical tests and direct confirmation of the biological data. In comparison with the approaches which predict the interactions based on the cliques, the overlap of the predictions is small. Similarly, the overlaps among the predicted sets of interactions derived from various complex sets are also small. Thus, every predicted set of interactions may complement and improve the quality of the original network data. Meanwhile, the predictions from the proposed method replenish protein-protein interactions associated with protein complexes using only the network topology.

## 1. Introduction

Protein-protein interactions (PPIs) contribute to the interpretation of cellular organization, processes, and functions. They also compose bigger molecules and protein complexes to perform molecular functions [1]. The deposition of PPIs has recently been enriched by high-throughput biological experiments [2]. Although the PPIs identified by such experiments are somehow reliable, they produce a number of false-positive and false-negative interactions [3], which subsequently influence the associated downstream tasks. Therefore, numerous computational approaches have been designed to predict and estimate PPIs based on the existing PPI datasets [4, 5]. The PPIs predicted with these approaches complement each other because they are based on different backgrounds of biological knowledge or hypotheses. The categories of the methodologies used for predicting PPIs differ among studies. For example, PPIs are classified by the structural, genomic, and biological contexts in reference [6]. PPIs used to detect protein complexes are always related to interactions derived from experimental technology of affinity purification [7]. Moreover, PPIs associated with protein complexes can be visualized with 3D structure data through the interface region on their surfaces. Structural approaches provide the physical details of the interactions at the protein interface that contributes to the protein complexes identification [6]. However, comparing with the approaches of genomic and biological contexts, the structural approaches tend to be more limited in terms of the scale because only a few proteins have 3D structures deposited in the Protein Data Bank (PDB) [8]. Despite the structural approaches, computational methods scarcely predict PPIs associated with protein complexes.

On the other hand, the known PPIs compose protein interaction networks. Many approaches are designed to predict PPIs and protein complexes based on the topology of the PPI networks [9]. They enjoy the advantages of simplicity and no extra information. The dense regions in the PPI networks are prone to be related to the functional modules and protein complexes [10]. For instance, cliques (maximal complete subnets) in PPI networks can be used to predict PPIs [11, 12]. Unfortunately, topological approaches to predicting PPIs have difficulties in identifying the PPIs associated with protein complexes. Therefore, predicting PPIs related to protein complexes based on protein interaction networks is significant for detecting the protein complexes.

**Figure 1:**
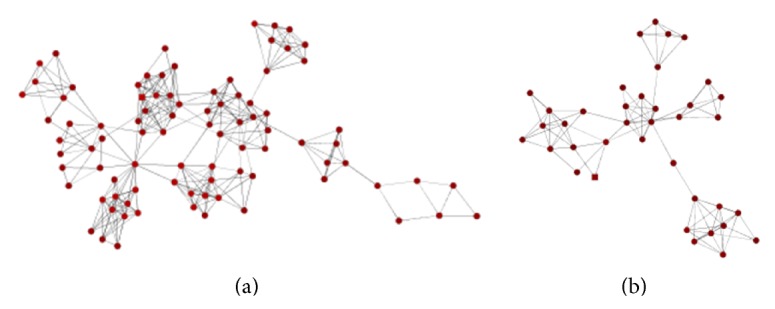
Two complexes detected by MCODE based on DIP dataset and composed of several subnets. Proteins between various subnets or within loosely connected subnets have the low possibility of the interaction.

Proteins in protein complexes tend to interact with each other [13]. There are also many complex detection algorithms based only on the protein interaction networks [9]. Thus, the detected protein complexes can be utilized to predict PPIs based only on the network topology; that is, the detection algorithms identify protein complexes, and then the PPIs are predicted among proteins in the complexes. However, there are two major problems predicting PPIs if the detected complexes are directly used. First, a protein complex may consist of several subnets and proteins between different subnets are not likely to interact. Second, proteins in an independent subnet of a complex may connect with each other loosely, instead of interacting with each other.  Figure 1 contains two protein complexes detected by the algorithm MCODE [14] based on the protein interaction network of yeast derived from DIP [15], which has the two problems mentioned above. Many detected protein complexes are located in dense regions of networks, while some may be loosely connected subnets.

According to the different structures of protein complexes, it is more feasible to predict the PPIs based on the densely partial subnets in the complexes, which always include areas of tight connection regardless of the differences in the size, number, and distribution of the topological structure. Therefore, we intend to disassemble the complexes when searching for the dense regions. A *k*-core which is a complete subnet composed of *k* nodes is used as a seed to disintegrate a complex with extension and pruning. The *k*-core is the local structure of topology and can ensure the dismemberment of various detected complexes. A protein complex is decomposed and pruned into several small subnets connected tightly by extended *k*-cores and the proteins in the complex of loose connections that are abandoned. Finally, the proteins in the subnets can be predicted to interact.

To validate the proposed methods of predicting PPIs, we choose three complex detection algorithms which found complexes highly different in the number, size, and topological distribution based on the DIP dataset from* Saccharomyces cerevisiae*. The predicted PPIs are evaluated using a statistical method based on the gold standard [16], and the results are satisfying by comparing them with the ones from the methods based on cliques. Besides, the predicted protein pairs are directly estimated with the BioGRID database [17], which collects numerous PPIs from different biological experiments, and a big overlap between them is found. Moreover, a predicted PPI is more reliable if it can be repeatedly predicted according to multiple complexes. There are small overlaps among the predicted PPI sets based on complexes derived from different complex detection algorithms. Small overlaps are obtained between our predicted PPIs and the ones from the clique methods. The predicted PPIs can complement deficient PPIs associated with protein complexes in protein interaction networks.

The remainder of this paper is organized as follows. In Section 2, we describe our method of getting PPI predictions. In Sections 2.2 and 2.3, we present the key steps of predicting PPIs by pruning complexes with the adaptive *k*-cores method and its improvement in a special condition. In Section 3, the results of our method are applied to predicting PPIs in the DIP dataset of yeast. Conclusions are drawn in Section 4.

## 2. Methods

**Figure 2:**
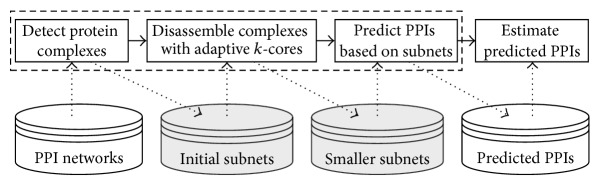
Flowchart of PPI prediction and estimation. Based on PPI networks, complex detection algorithms identify the initial regions of the PPI prediction. The adaptive *k*-cores method extracts the more accurate scope of the prediction. The PPIs are predicted in smaller subnets and are finally estimated with two methods.

In this section, we will present the method of predicting PPIs in three steps and introduce the methods of estimating the predicted PPIs (see Figure 2). There are many highly connected regions in a PPI network which tend to associate with functional modules or protein complexes. Identification of highly connected sets can be achieved using various techniques [18]. Initially, the known complex detection algorithms are used to find dense regions, and the proteins in these regions possibly interact with each other. To improve the interacting possibility of proteins in the dense subnets, we apply the adaptive *k*-cores method to dissemble these subnets into smaller parts in which the proteins connect with each other more tightly. Proteins within smaller subnets are predicted to interact if they do not interact with each other in the original network. Finally, the performance of predicted PPIs is estimated using two ways.

### 2.1. Detection Algorithms of Protein Complex

This paper selects three algorithms for detecting the protein complexes and finding the initial subnets in a PPI network, that is, MCODE [14], COACH [19], and NDComplex [20]. They identify the complexes based only on the network topology and the detected complexes are quite different in the structural features of subnets. MCODE detects complexes based on the weight of the seed node, that is, the local neighbor density of each node, to extend and cluster new nodes via selecting the high weight nodes. The number of detected complexes is small, but their sizes are large, and their topology distributions are loose. COACH identifies complexes using two steps: (1) core nodes are determined according to the neighbor relationship of the nodes and (2) the core nodes are extended to complexes by following the structure direction of the biological significance. The detected protein complexes are large in size and number but are connected loosely. NDComplex identifies complexes by extending the overlapping subnets. The detected complexes are large in size and number but have a relatively high density of subnets.

### 2.2. Complex Decomposition with Adaptive *k*-Cores Method

For a detected protein complex, a *k*-core in it is determined randomly as the extended seed firstly. And then the seed is expanded in the region of the complex until the set conditions cannot be satisfied. The subnet based on extended *k*-core is pruned from the complex. The nodes of the rest of the complex are treated as a new complex. Finally, the above process is performed repeatedly until no *k*-core is found and the nodes of the rest of the complex are abandoned.

**Figure 3:**
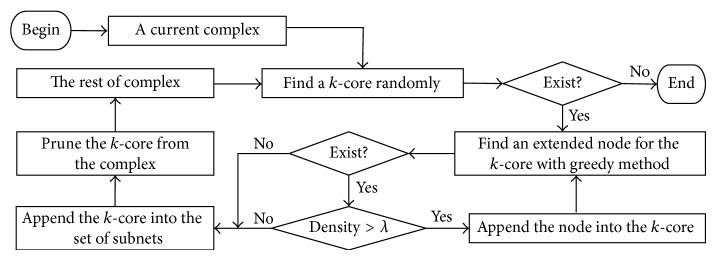
Flowchart of pruning a complex based on the adaptive *k*-cores method. First, a *k*-core is identified in the current complex. The process of pruning the complex ends if no *k*-core is found. Second, the *k*-core is extended with greedy method and is pruned from the complex when it is unsatisfied with the density threshold. Finally, the rest of complex is treated as the current one and the process of pruning complexes is repeated until a *k*-core cannot be found in the current complex.

The greedy method is introduced to extend the seed of the *k*-core. A node in the detected complex that has the maximum number of connections with the *k*-core is chosen and appended into the *k*-core. The density of the extended *k*-core is calculated by density = 2 *m*/*n*(*n* − 1), where *m* and *n* are the number of edges and nodes in the extended *k*-core, respectively. If the density is larger than a threshold *λ*, then extended *k*-core is set as the new seed and continues to be expanded. Otherwise, the expansion stops. See Figure 3 for the process used to predict PPIs.

The time complexity of the proposed method is analyzed as follows. For a protein complex, there are *n* protein nodes, *m* protein pairs, and* l k*-cores. Finding a *k*-core in the complex is *O*(*nml*) [21]. In the neighbors of the nodes of the *k*-core, finding a node that has the maximum number connected with the *k*-core is *O*(*n*). Calculating the subnet density is *O*(*n* ^2^). Predicting PPIs based on the subnets decomposed from the complex is also *O*(*n* ^2^). Therefore, the final time complexity is *O*(*nml* + *n* ^2^). In practice, the number of nodes in the protein complex, *n*, is not too large, and it will decrease after an extended *k*-core is found. Thus, the real processing time is very short.

**Figure 4:**
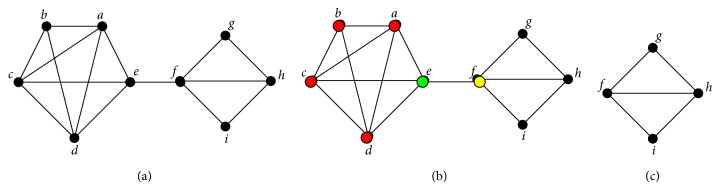
An example of decomposing a complex with *k*-cores.

Figure 4 shows an example of pruning a protein complex based on an extension with a 4-core seed.  Figure 4(a) represents a protein complex.  Figure 4(b) identifies a 4-core* abcd* that can extend node *e*.  Figure 4(c) shows that the subnet* abcde* is pruned and the rest of the subnet* fghi* is not extended and is therefore abandoned. Consequently, only one subnet* abcde* is obtained to participate in the PPI prediction from the complex, and the other parts of the complex are discarded.

### 2.3. Adjustment of Adaptive *k*-Cores Method in a Special Condition

There may be overlapping nodes among various protein complexes derived from a certain complex detection algorithm, such as algorithms COACH and NDComplex. The extended cores of COACH may have overlaps but its section is small. The overlaps among complexes derived from NDComplex are relatively large. So, various complexes are decomposed into small subnets and may predict the same PPIs. Therefore, we introduce a parameter of repetitive prediction, *h*. The initial value of *h* is one for every predicted PPI. If a predicted PPI is to be predicted again by another subnet, the value of *h* is increased by one for the corresponding PPI. A high *h* corresponds to multiple times for a predicted PPI with different complexes and can present a better possibility for the reliability of the predicted pairs.

### 2.4. Prediction of PPIs in Subnets

**Figure 5:**
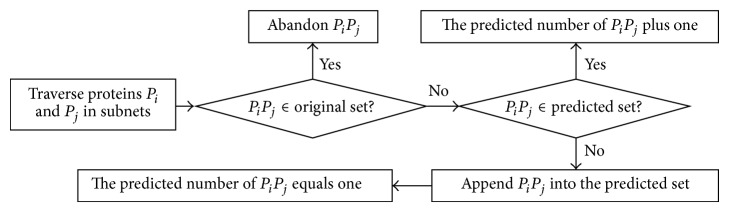
Flowchart of PPI prediction among subnets. The interactions are predicted in the subnets and the predicted numbers are recorded simultaneously.

A set of original interactions is built based on the PPIs of the network and is defined as the original set. A set of predictions is used to store the predicted PPIs and is defined as the predicted set which is initialized with null. We traverse every protein in a subnet and test the arbitrary proteins *P*_*i*_ and *P*_*j*_ to determine whether the interaction *P*_*i*_ *P*_*j*_ can be put in the predicted set (see Figure 5). Simultaneously, we count for every prediction. The interaction *P*_*i*_ *P*_*j*_ equals *P*_*j*_ *P*_*i*_ and only one interaction is determined. The process will be performed for every subnet derived from all complexes. The final predictions of PPI are all deposited in the predicted set. The time complexity is *O*(*n* ^2^) if the subnet contains *n* proteins.

### 2.5. Estimation of the Predicted PPIs

Two methods are proposed to estimate the predicted PPIs. The first estimation is a statistical method based on a likelihood ratio *L* [16]. In this method, Jansen et al. introduce a gold standard (GS) dataset which contains two reliable sets of PPIs, that is, a true positive set and a true negative set. *L* = (*P*_+_/*G*_+_)/(*P*_−_/*G*_−_), where *P*_+_ is the number of predicted PPIs contained in the true positive GS set, *P*_−_ is the number of predicted PPIs in the true negative GS set, *G*_+_ is the number of the true positive sets of GS, and *G*_−_ is the number of the true negative sets of GS. *G*_+_ and *G*_−_ are constant and equal to 8250 and 2705844, respectively. The predicting performance is good if most of predictions hit in the true positive set and a few of predictions drop in the true negative set. This method can overcome the biased assessment from the deficient samples between positives and negatives. Jansen et al. have set two thresholds of *L*, that is, 300 and 600 [16]. The value *L* of predicted PPIs is acceptable if *L* is more than the two thresholds. Of course, the larger *L* is the better.

The second way of the estimation is via a direct comparison with other records of biological experiments. There are many datasets from various biological experiments for yeast. Database BioGRID collects sufficient and reliable data in* Saccharomyces cerevisiae* from primary literature [17]. Therefore, we compare the predicted PPIs with the BioGRID dataset (version 3.2.98), which includes 319436 PPIs of yeast. The overlap rate between the PPI predictions and the PPIs in BioGRID is calculated. The predicted PPIs are prone to be true positive if they have the high percentage of hits in BioGRID.

## 3. Results

Among the methods of predicting PPIs derived from dense regions of PPI network, clique methods have the strictest topology so that they can obtain good performance of the PPI prediction. Therefore, we compare our methods with the clique methods and hope to obtain similar performance. The analysis of the predicting performance is presented in Section 3.1. In Section 3.2, we will test the influence on the performance via selecting the different values of the density threshold. In Section 3.3, we present the better performance of the adjustment method. The remainder sections of Results section will present the advantages of the proposed method. In Sections 3.4 and 3.5, we will present the differences of prediction sets among various methods so that our multiple sets of PPI prediction can complement PPI dataset together. Furthermore, we will present the correlation between PPI predictions and protein complexes in Section 3.6.

Our method of predicting PPIs associated with complexes is applied to a large-scale PPI network from the DIP dataset of yeast (version of 2010/6/14) [15]. DIP is generally acknowledged as an excellent data source containing PPIs determined experimentally. The dataset of the version contains 26,718 interactions. In order to adapt to various complex detection algorithms, the proteins of self-interacting and reduplicative interactions are deleted. Finally, a protein interaction network is achieved with 4,997 protein nodes and 23,233 interaction pairs from DIP database. Concurrently, we select the algorithms MCODE, COACH, and NDComplex to obtain the three sets of protein complex based on the DIP dataset, respectively. MCODE has four parameters, that is, the vertex weight percentage (VWP) which defines the density of the resulting complex, threshold of fluff, and two Boolean options (haircut and fluff). We aim to obtain large complexes which do not need to be postprocessed. Thus, we set VWP to 0.2, fluff to be false, and haircut to be true according to the application of the similar PPI network from MIPS [14] and obtain 50 protein complexes. For COACH, there is only one parameter, the threshold of the neighborhood affinity. The bigger the value of the threshold is, the bigger the overlaps among complex cores are obtained and the higher the *F*-measure of detected complexes is achieved in the threshold range between 0 and 0.1 [19]. Thus, this parameter is set to 0.05 and 274 protein complexes are obtained. NDComplex has four parameters, that is, *t*, *c*, *d*, and *s*. The first two represent the similarity threshold and the occurrence threshold during the computation of neighborhood density, respectively. The last two represent the subnet density in low and dense regions, respectively. They are set to 0.3, 3, 0.7, and 0.2 sequentially to get the best overall performance [20] and 1,184 complexes are predicted.

### 3.1. Performance of Our Method

According to the scales of detected protein complexes from the three complex sets, we set three sizes of the *k*-core to our method, that is, 4, 4, and 7, respectively. Generally, a subnet in a PPI network can be judged as a dense one when its density threshold exceeds 0.5 [22]. We adopt a tradeoff threshold 0.7 to judge dense subnets in this research [12]. And we will discuss the performances of selecting different thresholds in the next section. We get three prediction sets of PPIs, which are denoted by M, C, and N, respectively. There are many overlap sections between various complexes derived from NDComplex. Thus, we apply the adjustment of our algorithm; that is, we get a new predicted PPI set from N via selecting predictions of *h* > 1, and this set is denoted by N^+^. The detailed prediction sets of M, C, N, and N^+^ are listed in the Supplementary Material (available online at http://dx.doi.org/10.1155/2015/259157).

Meanwhile, we introduce two approaches of predicting PPIs based on cliques, Yu et al. [11] and Yang and Tang [12], to contrast with the performance of our algorithm. Yu's approach predicts PPIs based on protein interaction networks by completing the defective cliques, which is stricter and more reliable compared with the methods based on clustering subnets and functional classification in the protein interaction network [11]. Yang's approach gets PPI predictions based on clique extension and rule filtration of gene ontology, and this is more stable and reliable than predicting methods using only the network topology. Based on the DIP network, the predicted set of Yu's approach is denoted by YU, and the two prediction sets obtained by Yang's approach are denoted by CORE and ALL, respectively.

**Figure 6:**
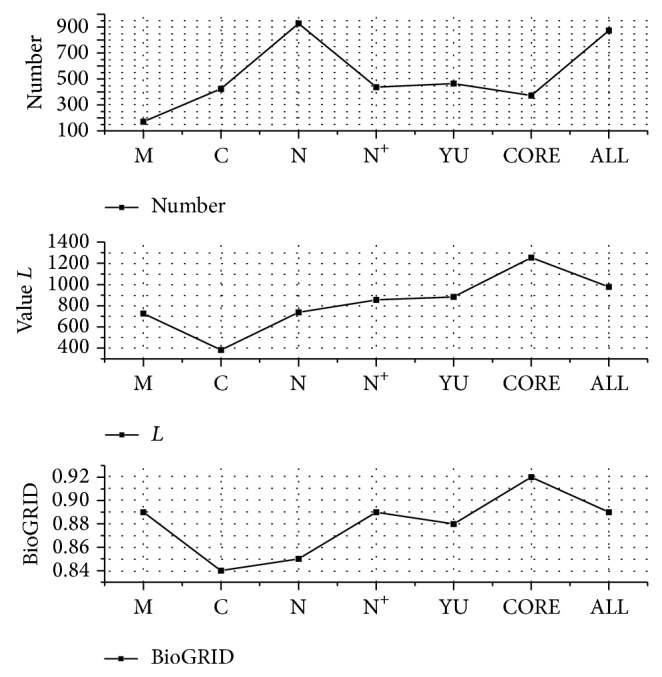
Performance of different prediction sets based on the number, statistical likelihood ratio *L*, and percentage hit in BioGRID of the PPI predictions.

The performance of various predicted PPIs is shown in Figure 6, which is estimated with the predicted number, likelihood value *L* of statistical significance, and hitting ratio of BioGRID validation, respectively. The number of N that is close to the number of ALL is the largest at 928, and the number of M is the smallest at 171. The others are close to each other. These predicted numbers are consistent with the numbers of protein complexes derived from various complex detection algorithms. All of the *L* values of predictions are acceptable based on the low standard. Most of them are close to YU, except for the predictions based on COACH set. The value *L* of the predictions based on algorithm COACH is the lowest. The complexes from COACH are large, and the distribution of nodes in complexes is prone to be very loose. This illustrates that the large and loose structures of complexes are not conductive to predicting PPIs. All of these predictions have the relatively high percentage of hitting in BioGRID. Although the protein complexes derived from different complex detection algorithms are diverse in their topological structure, our method of predicting PPIs based on them is stable and the performance is close to the methods of Yu and Yang.

### 3.2. Selection of Subnet Density Threshold

**Figure 7:**
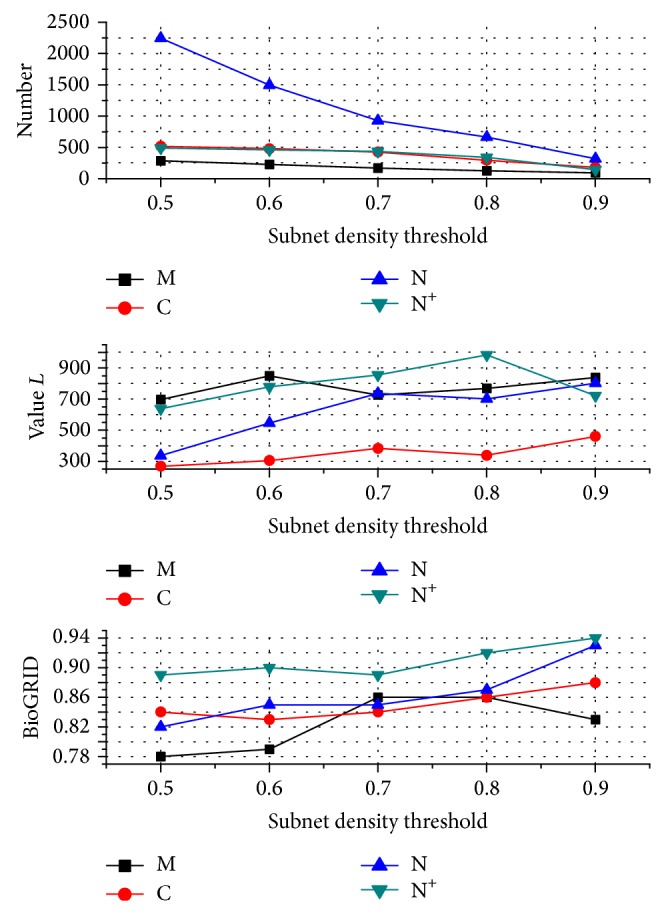
Performance of prediction sets based on the complex sets M, C, N, and N^+^ derived from the different *λ*. The tradeoff threshold 0.7 almost gets good performance based on the number, statistical likelihood ratio *L*, and percentage hit in BioGRID of the PPI predictions.

The values of the subnet density threshold result in different predicted sets. If the density of a subnet is 1, the subnet is the completely connected region. We choose five values of threshold *λ* between 0.5 and 0.9 to test the performance of the predicted PPIs (see Figure 7). The maximum number of the predictions derived from complex set N is 2245, the lowest value *L* of the predictions is 268 based on complex set C, and the lowest hitting ratio in BioGRID of the predictions is 78% based on complex set N. All of them are obtained when *λ* is 0.5. With the increase of *λ*, we almost get lower number of predictions, higher likelihood ratio *L* estimated with statistical significance, and higher hitting ratio in BioGRID. Higher threshold is conductive for generating more accurate quality of predictions but lower number of predictions, and vice versa. We achieve good performance when *λ* is 0.7 based on these three aspects. So, the tradeoff threshold 0.7 is recommended. We may also choose the *λ* value of 0.9 if we merely care about the quality of predicted PPIs and not about the quantity.

### 3.3. Estimation of the Adjustment Method

**Figure 8:**
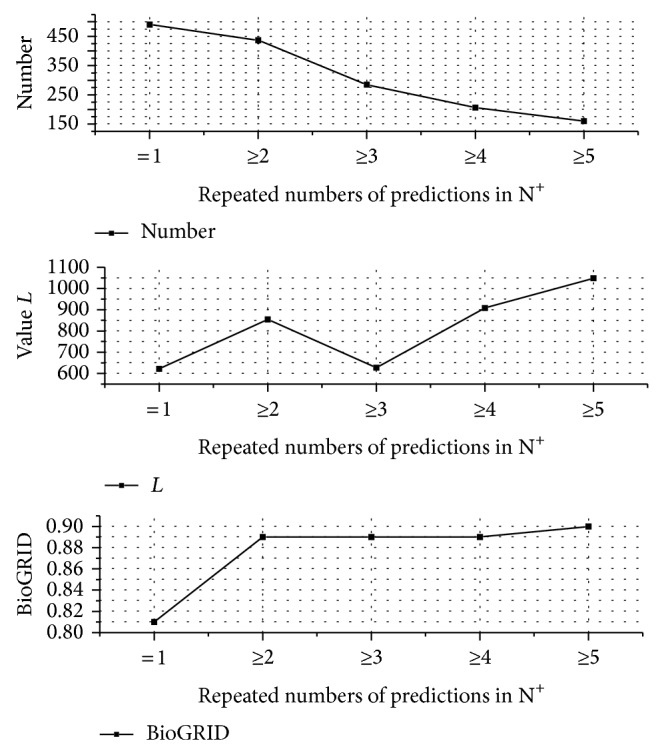
Estimation of predicted PPIs oriented from N^+^ based on the adjustment method. With the growth of the repeated number of PPI predictions, the number of the predictions decreases, and the value *L* and percentage of hitting in BioGRID are almost increasing.

The predicted PPIs based on the complexes from NDComplex have many repetitive ones. The maximum number of the repetitive predictions is 19. We estimate the effect of the repetitive number of predicted PPIs for the reliability of predictions (see Figure 8). The predicted set, N, contains 928 protein pairs of which 437 (nearly 50%) are predicted once. Predictions repeated more than five times are nearly 160. The value of *L* rises almost with the increase of the repetitive number. This is consistent with the hitting ratio in BioGRID. The higher repeated number obtains the better performance of PPI prediction when tolerating the lower number of PPI predictions. We obtain a tradeoff value of parameter *h* based on the prediction number, value *L*, and hitting ratio in BioGRID, that is, *h* > 1, to obtain more reliable predictions of PPI.

### 3.4. Comparison with Predictions Based on Clique Methods

Cliques in protein interaction networks also associate tightly with protein complexes. Therefore, we examine the relationships of the predictions from the cliques and detected complexes. There are 465 predicted PPIs based on Yu's method, 372 predictions in CORE, and 874 in ALL. We compare our predictions of PPIs with the ones oriented from Yu, CORE, and ALL, respectively (see Table 1). The overlap ratio between the two prediction sets is a percentage calculated with the division of the intersection and union of the two prediction sets. The common predictions between Yu and N^+^ are close to one-half. Except for predictions of Yu and N^+^, the overlap ratios of predictions are about one-third. This illustrates that our predicted PPIs are different compared with the ones derived from clique methods and can complement deficient interactions in PPI networks. Our method can improve PPI networks sequentially if more complexes detected by different algorithms are introduced.

### 3.5. Complement between the Predictions from Different Complexes

**Figure 9:**
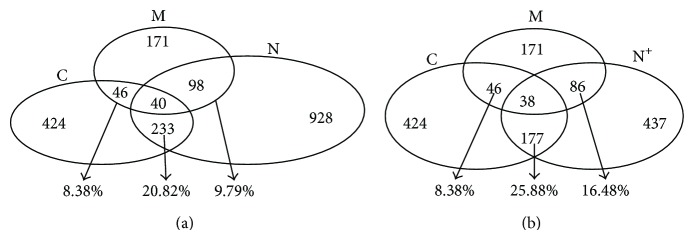
Overlap rates between predictions oriented from different complex sets. They are less than 30%, showing the good complementarity.

This section identifies the relationship between various predictions of PPIs based on different protein complex sets.  Figure 9(a) indicates the relationship between predicted sets M, C, and N. The three sets have 1186 predictions of PPIs. There are 40 common PPIs. The overlap section between C and N is the largest and has 233 common interactions (about 20%) between the two sets. This illustrates that the predictions based on different complex detection algorithms have good complementarities.  Figure 9(b) shows the relationship among predicted sets M, C, and N^+^. Only two protein pairs are absent from the common predictions of the three sets, which illustrates that the quality of the predictions of N^+^ is better than those of N. Therefore, the adjustment of our method can obtain more reliably predicted PPIs.

### 3.6. Association between Predicted PPIs and Complexes

The predictions of M, C, N, and N^+^ are annotated on the cellular components of gene ontology (GO) [23]. We identify predicted PPIs associated with protein complexes using semantic screening; that is, proteins in predictions must be included in the same GO term containing the word* complex*. The predicted protein pairs from different prediction sets related to complexes are about one-third (see Table 2). Because of the incompleteness of GO annotation and semantic screening, the real hitting ratio in complex of predictions may be higher in reality.

**Figure 10:**
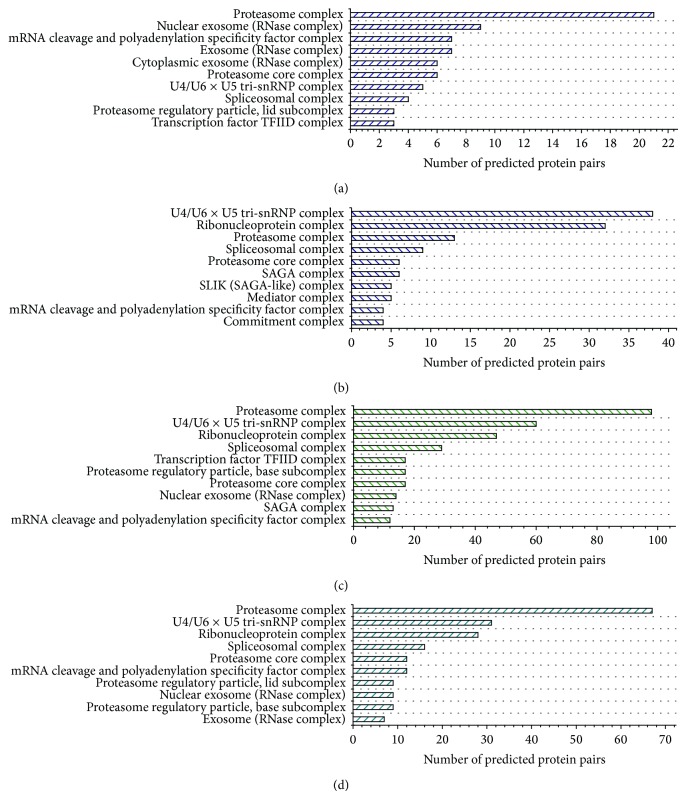
Four groups of top 10 complexes derived from GO annotation based on the predicted PPI sets corresponding to M, C, N, and N^+^, respectively. Here, the vertical axis represents the term of GO annotations and the horizontal axis represents the number of predicted PPIs.

Figure 10 shows the top 10 GO annotations of protein complexes corresponding to M, C, N, and N^+^, respectively. There are six types of protein complexes in the four collections of top 10 rankings, including proteasome complex, U4/U6 × U5 tri-snRNP, spliceosomal complex, transcription factor TFIID complex, proteasome core complex, and mRNA cleavage and polyadenylation specificity factor complex. The PPIs from different predictions focus on different complexes that are associated with the complex detection algorithms. The number of predictions between C and N^+^ is almost the same, but the hitting ratio in terms of protein complex has a wide gap. This is because the complexes derived from COACH are large and loose in the structure and some proteins in them are not likely to be in the same complex.

## 4. Conclusions

Various protein complex detection algorithms produce complexes having different features in terms of the number, size, and distribution of the nodes. Nevertheless, the method of decomposing complexes based on *k*-cores can identify the dense regions in complexes despite the topological structure of the complexes. This paper proposes a method of predicting PPIs that is adaptive to various complexes robustly and the predictions are reliable with the estimations. The predictions based on various complexes detected with different algorithms can complement each other, and they differ from the ones derived from the clique methods. Therefore, the predicted PPIs can supplement the deficient data of the protein interaction networks associated with the protein complexes. The improved networks contribute to detecting the protein complexes and studying the relationship of proteins in complexes.

## Supplementary Material

The Supplementary Material is described as follows. “The Supplementary Material contains four PPI sets which are predicted based on four groups of protein complexes derived from various complex detection algorithms.

## Figures and Tables

**Table 1:** Overlap ratio of predictions between our method and YU, CORE, and ALL, respectively.

Predicted set	∩YU	∪YU	O_1_	∩CORE	*∪*CORE	O_2_	∩ALL	∪ALL	O_3_
M	184	705	26%	51	492	10%	102	943	11%
C	94	542	17%	114	682	17%	226	1072	21%
N	344	1049	33%	197	1103	18%	432	1370	32%
N^+^	294	608	48%	137	672	20%	305	1006	30%

**Table 2:** Ratio of predictions hit in annotations of GO of protein complex.

Predicted set	Prediction number	Hitting in complex	Hitting ratio
M	171	63	36.84%
C	424	107	25.24%
N	928	282	30.39%
N^+^	437	164	37.52%
