# Supplementary material for: Prediction of Protein-Protein Interactions Related to Protein Complexes Based on Protein Interaction Networks
Source: Biomed Res Int. 2015 Feb 3;2015:259157. doi: 10.1155/2015/259157 (PMC4333188; doi:10.1155/2015/259157)
Supplement: Supplementary file 1 — The Supplementary Material is described as follows. “The Supplementary Material contains four PPI sets which are predicted based on four groups of protein complexes derived from various complex detection algorithms. [file 259157.f1.pdf]

Prediction of protein-protein interactions related to protein complexes based on protein interaction networks

Supplementary materials

Content

S1. PPI predictions based on the detected complexes derived from MCODE (M) …………1
S2. PPI predictions based on the detected complexes derived from COACH (C) …………2
S3. PPI predictions based on the detected complexes derived from NDComplex (N)………5
S4. PPIs predicted at least twice based on the prediction set N (N^+) ………………………20

S1. PPI predictions based on the detected complexes derived from MCODE (M)

[1] RNA14	YTH1	[2] RNA14	GLC7	[3] RNA14	PTI1
[4] PFS2	GLC7	[5] GLC7	PTI1	[6] YSH1	PTI1
[7] REF2	FIP1	[8] RPN5	RAD23	[9] RPT2	RPN8
[10] RPT2	RPN9	[11] RPT2	RPN7	[12] RPN12	UBP6
[13] PRE1	UBP6	[14] RPN8	RPN7	[15] UBP6	RAD23
[16] UBP6	RPN7	[17] RPN9	RPN7	[18] RAD23	RPN7
[19] RAD23	RPN11	[20] RPN7	RPN11	[21] DCP1	PAT1
[22] APC4	CDC16	[23] RPF2	NOP4	[24] MAK21	RIX1
[25] MAK21	TIF6	[26] RLP7	CIC1	[27] RLP7	HAS1
[28] RLP7	RIX1	[29] RLP7	YTM1	[30] RLP7	NOP7
[31] RLP7	NOC2	[32] CIC1	HAS1	[33] CIC1	RIX1
[34] CIC1	NOP7	[35] CIC1	TIF6	[36] HAS1	RIX1
[37] HAS1	YTM1	[38] HAS1	NOC2	[39] ERB1	RIX1
[40] RIX1	YTM1	[41] RIX1	NOP4	[42] RIX1	NOP7
[43] RIX1	NOC2	[44] RIX1	SSF1	[45] YTM1	NOC2
[46] YTM1	SSF1	[47] NOP4	TIF6	[48] NOP7	NOC2
[49] NOC2	TIF6	[50] SWD2	SHG1	[51] SWD2	SDC1
[52] RRP43	RRP6	[53] RRP43	SKI7	[54] RRP43	RRP42
[55] RRP43	MTR3	[56] RRP6	SKI7	[57] RRP6	RRP4
[58] RRP6	RRP42	[59] RRP6	MTR3	[60] SKI7	RRP42
[61] SKI7	MTR3	[62] SKI7	SRP1	[63] RRP4	MTR3
[64] RRP42	SRP1	[65] MTR3	SRP1	[66] SUI1	SUA7
[67] PRP40	PRP42	[68] ROX3	SRB6	[69] ROX3	MED2
[70] ROX3	SPT15	[71] SRB6	SPT15	[72] MED2	SPT15
[73] SRB4	SPT15	[74] GAL11	SPT15	[75] SPT15	MED7
[76] SNU114	PRP6	[77] SNU114	PRP3	[78] SNU114	SNU66
[79] PRP6	SNU66	[80] PRP3	SNU66	[81] ATP7	IPI3
[82] ATP17	IPI3	[83] ATP2	IPI3	[84] ATP18	IPI3
[85] ATP6	IPI3	[86] MUD1	LUC7	[87] MUD1	PRP4
[88] SMX2	PRP39	[89] SNU56	PRP39	[90] SNU56	CBC1
[91] SNU56	PRP4	[92] PRP39	CBC1	[93] PRP39	PRP4
[94] CBC1	PRP4	[95] SNU71	PRP4	[96] LUC7	PRP4
[97] NOP2	NOP15	[98] NOP15	MAK5	[99] MAK5	NSA2

[100] TAF9 TAF12 [101] TAF9 ADR1 [102] TAF9 NOT3
[103] TAF12 ADR1 [104] TAF12 TAF11 [105] TAF12 NOT3
[106] ADR1 TAF11 [107] ADR1 TAF13 [108] ADR1 NOT3
[109] TAF11 NOT3 [110] TAF11 TAF6 [111] TAF5 NOT3
[112] TAF13 NOT3 [113] NOT3 TAF6 [114] NOT5 POP2
[115] NOT2 POP2 [116] NOT2 CAF130 [117] SPT5 SPT16
[118] RTF1 SPT16 [119] SPT16 LEO1 [120] CSG2 AGP1
[121] PPA1 GSF2 [122] GSF2 YET1 [123] ARP4 EAF3
[124] YOP1 EMP24 [125] YOP1 ELO2 [126] YOP1 TPO3
[127] EMP24 SSH1 [128] EMP24 TPO3 [129] SSH1 TPO3
[130] RPB7 SIN4 [131] SIN4 RPB9 [132] SIN4 RPB4
[133] SIN4 RPB3 [134] RPB3 RPB1 [135] PRE10 PRE4
[136] PRE10 PRE9 [137] PRE10 PUP3 [138] PRE4 PRE9
[139] PRE4 PUP3 [140] PRE9 PUP3 [141] SEC28 SEC21
[142] SEC28 RET3 [143] SEC28 SEC26 [144] SEC21 RET3
[145] SEC21 SEC26 [146] RET3 SEC26 [147] GPI2 TSC13
[148] YPL264C ELO1 [149] YPL264C GAS3 [150] VPS8 VPS41
[151] UBP7 CHA1 [152] MYO3 CHA1 [153] MYO5 CHA1
[154] PMP1 TLG2 [155] PMP1 PMP2 [156] TLG2 YLL023C
[157] TLG2 ERV29 [158] TLG2 ELO3 [159] ERV29 ELO3
[160] TEF1 YGL081W [161] PRP21 YGL081W [162] CUS1 YGL081W
[163] VPH1 STV1 [164] ASF1 RLF2 [165] NUP1 SNO4
[166] NUP1 SNZ2 [167] NUP1 SNZ3 [168] RPT4 ATG12
[169] RPT4 RPN3 [170] RPT6 RPN3 [171] YGL004C ATG12

S2. PPI predictions based on the detected complexes derived from COACH (C)

[1] LEU2 VMA2 [2] VMA2 GAL1 [3] CPR1 SET3
[4] CPR1 ZDS1 [5] HST1 ZDS1 [6] SLA2 SLA1
[7] SLA2 SSC1 [8] SLA1 SSC1 [9] YSC84 SSC1
[10] CIC1 NOP7 [11] NOP4 BUD20 [12] NOP4 PAB1
[13] SHS1 BNI5 [14] BET1 SEC22 [15] PHO86 ALG1
[16] PHO86 CSG2 [17] PHO86 IFA38 [18] PHO86 GSF2
[19] PHO86 ELO1 [20] PHO86 SRP102 [21] ALG1 SUR2
[22] ALG1 CSG2 [23] ALG1 AVT4 [24] ALG1 GSF2
[25] ALG1 ELO1 [26] ALG1 SRP102 [27] ALG1 YPC1
[28] GPI8 CSG2 [29] GPI8 EMP24 [30] GPI8 YPL264C
[31] GPI8 AVT4 [32] GPI8 ERG25 [33] GPI8 COS8
[34] GPI2 TSC13 [35] GPI2 EMP24 [36] GPI2 FET3
[37] SUR2 CSG2 [38] SUR2 TSC13 [39] SUR2 AVT4
[40] SUR2 ERG25 [41] SUR2 COS8 [42] SUR2 GAS3
[43] SUR2 YPC1 [44] SUR2 FET3 [45] CSG2 EMP24
[46] CSG2 AVT4 [47] CSG2 ERG25 [48] CSG2 COS8
[49] CSG2 YPC1 [50] CSG2 FET3 [51] TSC13 YPC1
[52] TSC13 FET3 [53] IFA38 ELO2 [54] IFA38 SRP102

[55] IFA38 PHO88 [56] IFA38 ELO3 [57] IFA38 YET1
[58] EMP24 YPL264C [59] EMP24 AVT4 [60] EMP24 COS8
[61] EMP24 ELO1 [62] EMP24 YPC1 [63] EMP24 FET3
[64] YPL264C AVT4 [65] YPL264C COS8 [66] YPL264C ELO1
[67] YPL264C GAS3 [68] YPL264C YPC1 [69] AVT4 COS8
[70] AVT4 GAS3 [71] AVT4 FET3 [72] ERG25 COS8
[73] ERG25 ELO1 [74] ERG25 FET3 [75] GSF2 ELO1
[76] GSF2 ELO2 [77] GSF2 SRP102 [78] GSF2 PHO88
[79] GSF2 YET1 [80] COS8 GAS3 [81] COS8 FET3
[82] ELO1 YPC1 [83] ELO2 SRP102 [84] ELO2 PHO88
[85] ELO2 ELO3 [86] ELO2 YET1 [87] SRP102 PHO88
[88] SRP102 ELO3 [89] SRP102 YET1 [90] GAS3 YPC1
[91] GAS3 FET3 [92] PHO88 ELO3 [93] PHO88 YET1
[94] ELO3 YET1 [95] YPC1 FET3 [96] HSP60 TUB2
[97] PFK2 TUB2 [98] YNL132W NOP1 [99] YNL132W UTP20
[100] KRR1 UTP22 [101] NOP1 UTP20 [102] RPN12 YGL004C
[103] RPN12 UBP6 [104] RPN12 ATG12 [105] RPT6 RPN8
[106] RPT6 RPN9 [107] RPT6 RPN3 [108] PRE1 YGL004C
[109] PRE1 UBP6 [110] PRE1 ATG12 [111] YGL004C UBP6
[112] YGL004C ATG12 [113] RPN8 ATG12 [114] RPN8 RPN3
[115] UBP6 ATG12 [116] ATG12 RPN9 [117] RPN9 RPN3
[118] LEU2 TPD3 [119] SPP381 SMX3 [120] SPP381 SMX2
[121] RSE1 PRP11 [122] RSE1 PRP21 [123] SMX3 SMX2
[124] SMX3 PRP11 [125] SMX3 PRP21 [126] CIC1 HAS1
[127] CIC1 PUF6 [128] CIC1 TIF6 [129] HAS1 PUF6
[130] HAS1 YTM1 [131] PUF6 ERB1 [132] PUF6 YTM1
[133] PUF6 NOP7 [134] NUP145 NUP133 [135] PPH1 RAD59
[136] ARP2 RAD59 [137] LEU2 CDC15 [138] YKU80 CDC15
[139] SEC63 ELO3 [140] UTP18 NOP7 [141] UTP22 NOP7
[142] NOP7 CKB1 [143] STT3 SWP1 [144] RPN10 PPH22
[145] PRE6 PRE9 [146] PRE6 PUP3 [147] PRE6 PRE5
[148] PRE9 PUP3 [149] PRE9 PRE5 [150] PUP3 PRE5
[151] RNA14 YTH1 [152] RNA14 PTI1 [153] CFT1 DBP4
[154] DBP4 MPE1 [155] DBP4 YTH1 [156] YSH1 PTI1
[157] REF2 FIP1 [158] VMA2 VMA8 [159] NOT5 SSN2
[160] NOT5 POP2 [161] SSN2 NOT4 [162] SSN2 NOT3
[163] SSN2 CAF130 [164] NOT2 POP2 [165] NOT2 NOT3
[166] NOT2 CAF130 [167] NOT3 CCR4 [168] LSB3 RVS167
[169] LSB3 YSC84 [170] GCN4 TAF12 [171] GCN4 SPT7
[172] GCN4 TAF10 [173] GCN4 TAF13 [174] TAF9 TAF12
[175] TAF9 SPT15 [176] TAF12 SPT7 [177] TAF12 NGG1
[178] TAF12 ADA2 [179] SPT7 TAF1 [180] SPT7 SPT15
[181] SPT7 TAF13 [182] NGG1 TAF10 [183] NGG1 TAF13
[184] SPT15 TAF10 [185] SPT15 TAF13 [186] TAF13 ADA2

[187] SED5 NYV1 [188] VMA4 TFP1 [189] ROX3 SRB6
[190] ROX3 MED2 [191] ROX3 GAL4 [192] ROX3 ADA2
[193] SSN3 GAL11 [194] SSN3 MED8 [195] SSN3 MED6
[196] SSN3 SRB2 [197] SSN3 MED7 [198] SSN3 ADA2
[199] MED2 GAL4 [200] MED2 ADA2 [201] GAL11 ADA2
[202] MED8 MED6 [203] MED8 GAL4 [204] MED8 ADA2
[205] SRB5 MED6 [206] SRB5 ADA2 [207] MED6 SRB2
[208] MED6 GAL4 [209] MED6 ADA2 [210] GAL4 MED7
[211] HTZ1 ESA1 [212] SWR1 TRA1 [213] SWR1 ESA1
[214] SWR1 EPL1 [215] ACT1 TRA1 [216] TRA1 RVB2
[217] ESA1 RVB2 [218] RVB2 EPL1 [219] STI1 GAL1
[220] NUP100 NUP60 [221] NUP60 PSE1 [222] PSE1 NUP2
[223] SLA1 BBC1 [224] BBC1 RVS167 [225] RPB2 RPB8
[226] RPB3 RPB1 [227] RPB4 RPB8 [228] FPR1 RPC5
[229] NNF1 SPC24 [230] SPC24 NUF2 [231] PFK2 KAR2
[232] YKU80 KAR2 [233] LSM3 PRP6 [234] LSM3 SMD3
[235] LSM2 BRR2 [236] LSM2 SNU114 [237] LSM2 PRP6
[238] LSM2 PRP4 [239] BRR2 LSM6 [240] BRR2 LSM7
[241] BRR2 LSM1 [242] BRR2 LSM5 [243] SNU114 LSM6
[244] SNU114 PRP6 [245] SNU114 LSM4 [246] LSM6 PRP6
[247] LSM6 PRP4 [248] LSM7 PRP31 [249] LSM7 PRP8
[250] LSM7 SMD3 [251] PRP31 LSM4 [252] PRP31 LSM1
[253] PRP31 LSM5 [254] PRP6 LSM4 [255] PRP6 LSM1
[256] PRP8 LSM4 [257] PRP8 LSM1 [258] PRP8 LSM5
[259] LSM4 PRP4 [260] LSM1 SMD3 [261] LSM1 PRP4
[262] SUI1 SUA7 [263] PDC1 GCD6 [264] PDC1 GCD7
[265] PDC1 SUI3 [266] GCD11 GCD2 [267] GCD11 SUI3
[268] SUI2 GCD7 [269] GCD2 SUI3 [270] YKU80 RPC5
[271] RPC5 MKK2 [272] LSM3 SNU66 [273] SNU114 PRP3
[274] SNU114 SNU66 [275] LSM7 PRP3 [276] PRP6 SNU66
[277] PRP3 SNU66 [278] MUD1 SMX3 [279] MUD1 SMD2
[280] MUD1 LUC7 [281] MUD1 SMD3 [282] SMX3 SNP1
[283] SMX3 NAM8 [284] SMX3 YHC1 [285] SMX3 CBC2
[286] PRP40 SMD2 [287] PRP40 CBC2 [288] PRP40 SMD3
[289] PRP40 PRP42 [290] SMX2 NAM8 [291] SMX2 CBC2
[292] SMX2 PRP42 [293] SMD2 SNP1 [294] SMD2 PRP42
[295] SNP1 SMD3 [296] YHC1 CBC2 [297] YHC1 SMD3
[298] CBC2 SMD3 [299] CBC2 PRP42 [300] SMD3 PRP42
[301] HSP60 HSP26 [302] RFA3 MSH2 [303] MSH2 RAD52
[304] ATP17 HRR25 [305] HRR25 ATP6 [306] PFK2 GAL1
[307] ARP2 GAL1 [308] GPI2 PMP2 [309] PMP2 YPL264C
[310] PMP2 CAN1 [311] PMP2 SPC1 [312] YPL264C CAN1
[313] YPL264C SPC1 [314] LSM3 DCP2 [315] LSM3 EDC3
[316] DCP2 LSM6 [317] DCP2 LSM7 [318] DCP2 SMD3

[319] DCP2 LSM5 [320] DCP1 SMD3 [321] DCP1 PAT1
[322] LSM6 EDC3 [323] LSM7 EDC3 [324] EDC3 LSM4
[325] EDC3 LSM1 [326] EDC3 SMD3 [327] EDC3 PAT1
[328] EDC3 LSM5 [329] SMD3 PAT1 [330] RPF2 MAK5
[331] RPF2 NOP4 [332] RPF2 CKA1 [333] MAK21 NOG1
[334] MAK21 NSA2 [335] MAK21 TIF6 [336] MAK21 CKA1
[337] CIC1 RLP7 [338] CIC1 NOG1 [339] CIC1 NSA2
[340] RLP7 HAS1 [341] RLP7 NOP2 [342] RLP7 YTM1
[343] RLP7 NOP7 [344] RLP7 NOG1 [345] RLP7 CKA1
[346] HAS1 NOG1 [347] NOP2 NOP15 [348] YTM1 NOG1
[349] YTM1 NSA2 [350] YTM1 SSF1 [351] YTM1 CKA1
[352] NOP15 MAK5 [353] NOP15 SSF1 [354] NOP15 CKA1
[355] MAK5 NOP7 [356] MAK5 NSA2 [357] MAK5 TIF6
[358] MAK5 CKA1 [359] NOP4 NSA2 [360] NOP4 TIF6
[361] NOP7 NOG1 [362] NOP7 NSA2 [363] NSA2 SSF1
[364] NSA2 CKA1 [365] TIF1 GAL1 [366] SGE1 YHR140W
[367] SGE1 GPI2 [368] SGE1 TSC13 [369] SGE1 YPL264C
[370] SGE1 CAN1 [371] SGE1 GAS3 [372] ERP4 IFA38
[373] ERP4 GSF2 [374] ERP4 PHO88 [375] ERP4 ELO3
[376] ERP4 YET1 [377] TSC13 CAN1 [378] CAN1 GAS3
[379] SGE1 PHO86 [380] SGE1 ERG25 [381] SGE1 LAC1
[382] SGE1 NSG1 [383] GPI2 NSG1 [384] ERG25 NSG1
[385] LAC1 GSF2 [386] LAC1 NSG1 [387] BSD2 SPC1
[388] CDC4 MET30 [389] RPF2 IPI3 [390] NSA2 IPI3
[391] BSD2 YHR140W [392] BSD2 CSG2 [393] BSD2 ERG25
[394] BSD2 CAN1 [395] GPI2 POM34 [396] ERG25 POM34
[397] ERG25 CAN1 [398] POM34 CAN1 [399] POM34 GAS3
[400] CAN1 YPC1 [401] VPS8 VPS41 [402] RPB5 RPC3
[403] RPC3 RPB8 [404] BSD2 GPI8 [405] BSD2 AVT4
[406] BSD2 COS8 [407] IFA38 POM34 [408] AVT4 POM34
[409] POM34 COS8 [410] GPM1 HSP26 [411] MYO3 UBA1
[412] MYO5 UBA1 [413] ARP2 UBA1 [414] SLT2 SEC27
[415] MET30 UFD2 [416] HRT1 UFD2 [417] ATG12 SRP1
[418] APC4 CDC16 [419] KAP123 PAB1 [420] ARP4 SIR3
[421] ARP4 CKA1 [422] SIR3 CKA1 [423] HTB1 CKA1
[424] YCK1 TPK1

S3. PPI predictions based on the detected complexes derived from NDComplex (N)

[1] RPN5 RAD23 [2] RPN12 YGL004C [3] RPN12 UBP6
[4] RPN12 ATG12 [5] PRE1 YGL004C [6] PRE1 UBP6
[7] PRE1 ATG12 [8] YGL004C UBP6 [9] YGL004C ATG12
[10] YGL004C RAD23 [11] UBP6 ATG12 [12] UBP6 RAD23
[13] UBP6 RPN7 [14] ATG12 RAD23 [15] RAD23 RPN7
[16] RAD23 RPN11 [17] RPN7 RPN11 [18] LSM3 SMD3

[19] DCP1 PRP24 [20] DCP1 SMD3 [21] DCP1 PAT1

[22] LSM7 SMD3 [23] PRP24 LSM4 [24] PRP24 LSM1

[25] PRP24 SMD3 [26] PRP24 PAT1 [27] LSM1 SMD3

[28] SMD3 PAT1 [29] PHO86 ALG1 [30] PHO86 TPO1

[31] PHO86 IFA38 [32] PHO86 GSF2 [33] ALG1 GSF2

[34] GPI8 TPO1 [35] GPI8 EMP24 [36] GPI8 YPL264C

[37] GPI8 ERG25 [38] GPI2 TPO1 [39] GPI2 TSC13

[40] GPI2 EMP24 [41] TPO1 TSC13 [42] TPO1 EMP24

[43] TPO1 YPL264C [44] IFA38 ELO2 [45] IFA38 PHO88

[46] IFA38 ELO3 [47] IFA38 YET1 [48] EMP24 YPL264C

[49] YPL264C GAS3 [50] GSF2 ELO2 [51] GSF2 PHO88

[52] GSF2 YET1 [53] ELO2 PHO88 [54] ELO2 ELO3

[55] ELO2 YET1 [56] PHO88 ELO3 [57] PHO88 YET1

[58] ELO3 YET1 [59] SUI1 SUA7 [60] TIF34 HCR1

[61] SUA7 HCR1 [62] HCR1 NIP1 [63] NOT2 CAF16

[64] SSN3 CAF40 [65] SSN3 CAF16 [66] CAF40 CAF16

[67] CAF16 NOT1 [68] PHO86 STE24 [69] PHO86 ELO1

[70] ALG1 STE24 [71] ALG1 AVT4 [72] ALG1 ELO1

[73] ALG1 YPC1 [74] BSD2 YHR140W [75] BSD2 GPI8

[76] BSD2 AVT4 [77] BSD2 ERG25 [78] BSD2 COS8

[79] GPI8 AVT4 [80] GPI8 COS8 [81] TSC13 YPC1

[82] IFA38 STE24 [83] STE24 ELO2 [84] STE24 PHO88

[85] STE24 ELO3 [86] STE24 YET1 [87] AVT4 COS8

[88] AVT4 GAS3 [89] ERG25 COS8 [90] ERG25 ELO1

[91] COS8 GAS3 [92] ELO1 YPC1 [93] GAS3 YPC1

[94] BOS1 GPT2 [95] SEC22 GPT2 [96] SEC21 GPT2

[97] RET1 GPT2 [98] MUD1 SMD2 [99] MUD1 LUC7

[100] PRP40 SNU56 [101] PRP40 SMD2 [102] PRP40 CBC2

[103] PRP40 PRP42 [104] SMX2 NAM8 [105] SMX2 CBC2

[106] SMX2 PRP42 [107] SNU56 SMD2 [108] SNU56 CBC1

[109] SNU56 PRP42 [110] SMD2 SNP1 [111] SMD2 PRP42

[112] YHC1 CBC2 [113] CBC2 PRP42 [114] LSM3 DCP2

[115] KEM1 DCP1 [116] KEM1 LSM6 [117] KEM1 LSM7

[118] KEM1 PRP24 [119] KEM1 LSM1 [120] KEM1 LSM5

[121] DCP2 LSM6 [122] DCP2 LSM7 [123] DCP2 PRP24

[124] DCP2 LSM5 [125] DCP2 SMD3 [126] GCN4 SPT7

[127] GCN4 TAF10 [128] TAF9 SPT15 [129] SPT7 TAF1

[130] SPT7 SPT15 [131] NGG1 TAF10 [132] SPT15 TAF10

[133] YHR140W AGP1 [134] TSC13 AGP1 [135] TSC13 SHR3

[136] AGP1 AVT4 [137] AGP1 COS8 [138] AGP1 YPC1

[139] ELO2 SHR3 [140] SHR3 PHO88 [141] SHR3 ELO3

[142] SHR3 YET1 [143] BSD2 EMP24 [144] BSD2 YPL264C

[145] BSD2 CAN1 [146] GPI8 CAN1 [147] GPI2 NSG1

[148] TSC13 CAN1 [149] EMP24 CAN1 [150] EMP24 NSG1

[151] YPL264C CAN1 [152] YPL264C NSG1 [153] ERG25 CAN1
[154] ERG25 NSG1 [155] CAN1 GAS3 [156] GCN4 TAF12
[157] GCN4 TAF13 [158] TAF9 TAF12 [159] TAF12 ADA2
[160] SPT15 TAF13 [161] TAF13 ADA2 [162] WBP1 YHR140W
[163] WBP1 GPI8 [164] WBP1 ERG25 [165] WBP1 GAS3
[166] ALG1 SUR2 [167] SUR2 ERG25 [168] SUR2 GAS3
[169] PHO86 CSG2 [170] ALG1 CSG2 [171] GPI8 CSG2
[172] CSG2 COS8 [173] COS8 CAN1 [174] TAF5 GCN5
[175] TAF5 UBP8 [176] UBP8 TAF10 [177] UBP8 TAF6
[178] UBP8 ADA2 [179] ALG1 AGP1 [180] ALG1 LAC1
[181] BSD2 AGP1 [182] YHR140W LAC1 [183] GPI8 AGP1
[184] AGP1 YPL264C [185] GSF2 LAC1 [186] LAC1 ELO2
[187] LAC1 NSG1 [188] NSG1 YET1 [189] PHO86 BAP3
[190] GPI2 BAP3 [191] SMX2 BRR2 [192] SMX2 PRP8
[193] SMX2 LSM8 [194] SNU114 PRP6 [195] SNU114 SMB1
[196] SNU114 PRP3 [197] PRP6 SMB1 [198] PRP8 SMD1
[199] LSM8 SMD1 [200] SMB1 PRP3 [201] SMB1 PRP4
[202] PRP3 SMD1 [203] SMD1 PRP4 [204] RSE1 PRP43
[205] RSE1 SNU114 [206] PRP8 PRP45 [207] SPP381 PRP8
[208] SPP381 LSM8 [209] SPP381 SMD3 [210] SNU114 SNU66
[211] SNU114 SNU23 [212] PRP31 SNU23 [213] PRP6 SNU66
[214] PRP6 SNU23 [215] PRP8 SNU23 [216] LSM8 SNU23
[217] PRP3 SNU66 [218] PRP3 SNU23 [219] SNU66 SNU23
[220] RPN10 PPH22 [221] RPN5 PRE4 [222] RPN5 PPH22
[223] RPN5 PUP3 [224] RPN5 PRE8 [225] PRE4 PUP3
[226] PRE4 PRE5 [227] PUP3 PRE5 [228] LSM3 EDC3
[229] EDC3 LSM4 [230] EDC3 LSM1 [231] EDC3 PAT1
[232] RRP43 RRP6 [233] RRP43 SKI7 [234] RRP43 RRP42
[235] RRP6 SKI7 [236] RRP6 RRP4 [237] RRP6 RRP42
[238] SKI7 RRP42 [239] SKI7 SRP1 [240] RRP42 SRP1
[241] YNL132W NOP1 [242] UTP7 SOF1 [243] KRR1 UTP22
[244] SOF1 UTP22 [245] TIF5 CKB2 [246] SUI1 CKB2
[247] TIF34 CKB2 [248] SUA7 CKB2 [249] CTF8 POL30
[250] CTF18 POL30 [251] LSM3 SMX2 [252] LSM3 PRP6
[253] PHO86 AQY1 [254] PHO86 TPO3 [255] ALG1 AQY1
[256] GPI8 TPO3 [257] AQY1 GPI2 [258] AQY1 TSC13
[259] AQY1 IFA38 [260] AQY1 GAS3 [261] GPI2 TPO3
[262] TSC13 TPO3 [263] ERG25 TPO3 [264] PHO86 SLY41
[265] YHR140W SLY41 [266] ELO1 SLY41 [267] ELO2 SLY41
[268] BOS1 DSL1 [269] SEC27 DSL1 [270] SEC22 DSL1
[271] DSL1 SEC21 [272] SAM3 YHR140W [273] SAM3 TSC13
[274] SAM3 GAS3 [275] ALG1 GIT1 [276] GIT1 GPI8
[277] GIT1 YHR140W [278] GIT1 TSC13 [279] GIT1 IFA38
[280] GIT1 GSF2 [281] GIT1 ELO2 [282] GIT1 GAS3

[283] GIT1 NSG1 [284] GIT1 FET3 [285] TSC13 FET3
[286] GAS3 FET3 [287] NSG1 FET3 [288] TAF9 SPT20
[289] TAF1 SPT20 [290] TAF5 SPT20 [291] SPT20 TAF6
[292] GCN4 ADR1 [293] TAF9 ADR1 [294] NGG1 ADR1
[295] RNA14 SWD2 [296] RNA14 YTH1 [297] RNA14 GLC7
[298] RNA14 PTI1 [299] SWD2 DBP4 [300] SWD2 PCF11
[301] SWD2 PAP1 [302] SWD2 YTH1 [303] SWD2 PTI1
[304] SWD2 FIP1 [305] CFT1 DBP4 [306] DBP4 PCF11
[307] DBP4 MPE1 [308] DBP4 YTH1 [309] PCF11 PFS2
[310] PCF11 YTH1 [311] PCF11 GLC7 [312] PCF11 REF2
[313] PCF11 PTI1 [314] PCF11 FIP1 [315] PFS2 GLC7
[316] GLC7 PTI1 [317] YSH1 PTI1 [318] REF2 FIP1
[319] GIN4 HSP26 [320] BNI5 SHS1 [321] BNI5 HSP26
[322] CDC3 HSP26 [323] SPT7 ADR1 [324] SIN4 SRB2
[325] ROX3 SRB6 [326] ROX3 MED2 [327] RGR1 SRB2
[328] DCP2 RPS23A [329] DCP2 RPS28B [330] EDC3 RPS23A
[331] RPS23A RPS28B [332] RRP43 MTR3 [333] RRP6 MTR3
[334] SKI7 MTR3 [335] RRP4 MTR3 [336] MTR3 SRP1
[337] RPN10 RPT5 [338] RPT2 RPT4 [339] RPT2 RPT6
[340] RPT2 RPN8 [341] RPT2 RPT5 [342] RPT2 RPN9
[343] RPT2 RPN3 [344] RPT4 PRE1 [345] RPT4 RPN8
[346] RPT4 RPN9 [347] RPT4 RAD23 [348] RPT4 RPN3
[349] RPT4 RPN11 [350] RPT6 RPN8 [351] RPT6 RPT5
[352] RPT6 RPN9 [353] RPT6 RPN3 [354] RPN8 RPT5
[355] RPN8 RPN3 [356] RPT5 UBP6 [357] RPT5 RPN9
[358] RPT5 RPN3 [359] RPT5 RPN11 [360] RPN9 RPN3
[361] SSN2 GAL11 [362] SRB6 SFL1 [363] MED2 SFL1
[364] SFL1 GAL11 [365] SFL1 MED7 [366] VID21 HTZ1
[367] VID21 ARP4 [368] VID21 ACT1 [369] VID21 TRA1
[370] VID21 RVB2 [371] SWC4 SAP185 [372] HTZ1 ESA1
[373] HTZ1 SAP185 [374] ACT1 TRA1 [375] TRA1 RVB2
[376] ESA1 RVB2 [377] SAP185 RVB2 [378] SAP185 YAF9
[379] RVB2 EPL1 [380] RPB7 IKI3 [381] RPB5 TFG1
[382] RPB9 IKI3 [383] RPB2 TFG1 [384] TFG1 RPB4
[385] TFG1 IKI3 [386] TFG1 RPB1 [387] RPB3 RPB1
[388] RPN10 PRE10 [389] RPN10 POC4 [390] PRE6 PRE10
[391] PRE6 PRE9 [392] PRE6 PRE5 [393] PRE10 PRE9
[394] PRE10 PRE8 [395] PRE1 POC4 [396] PPH22 POC4
[397] PRE9 PRE5 [398] POC4 PRE2 [399] SPP381 LSM5
[400] BRR2 LSM5 [401] PRP31 LSM5 [402] PRP8 LSM5
[403] PRP3 LSM5 [404] SNU23 LSM5 [405] YHR140W YHL042W
[406] YHR140W SPC1 [407] YHL042W ELO1 [408] GPI8 SPC1
[409] TSC13 SPC1 [410] AGP1 SPC1 [411] YPL264C SPC1
[412] SPC1 GAS3 [413] RPB5 RPC17 [414] RPB5 RPC2

[415] RPC17 RPC2 [416] RPC2 RPB8 [417] RPB2 RPB8
[418] RPB4 RPB8 [419] RPB8 IKI3 [420] SMB1 DIB1
[421] DIB1 SMD1 [422] PHO86 PFA4 [423] ALG1 PFA4
[424] GPI2 FET3 [425] SUR2 FET3 [426] ERG25 FET3
[427] ELO2 PFA4 [428] PFA4 PHO88 [429] PFA4 YET1
[430] SWD2 SHG1 [431] SWD2 SDC1 [432] SET3 CPR1
[433] ZDS1 CPR1 [434] ZDS1 HST1 [435] ARC18 MYO5
[436] ARC15 MYO5 [437] ARC35 MYO5 [438] CDC28 ORC5
[439] CDC28 ORC4 [440] CDC28 ORC3 [441] RRP43 RRP40
[442] RRP6 RRP40 [443] RRP4 RRP40 [444] RRP40 CSL4
[445] RRP40 SRP1 [446] MUD1 SRP1 [447] PRP40 SRP1
[448] SMX2 SRP1 [449] YHC1 SRP1 [450] SNU71 SRP1
[451] LUC7 SRP1 [452] CDC27 APC9 [453] APC4 CDC16
[454] APC1 APC9 [455] APC9 CDC26 [456] APC9 DOC1
[457] DNL4 ERB1 [458] DNL4 GLC7 [459] DNL4 NOP6
[460] RPF2 GLC7 [461] CIC1 NOP7 [462] CIC1 TIF6
[463] NOP2 NOP15 [464] NOP2 GLC7 [465] NOP15 GLC7
[466] NOP15 NOP6 [467] GLC7 NOP6 [468] GLC7 TIF6
[469] SIN4 SRB8 [470] RGR1 SRB8 [471] SRB8 SRB2
[472] MAK21 NOP6 [473] MAK21 TIF6 [474] MAK21 CKA1
[475] RPF2 NOP4 [476] RPF2 CKA1 [477] CIC1 HAS1
[478] HAS1 YTM1 [479] YTM1 SSF1 [480] YTM1 CKA1
[481] NOP15 SSF1 [482] NOP15 CKA1 [483] NOP4 NOP6
[484] NOP4 TIF6 [485] NOP6 SSF1 [486] NOP6 CKA1
[487] OST3 PKC1 [488] OST4 PKC1 [489] OST5 PKC1
[490] PKC1 OST2 [491] STT3 SWP1 [492] SMX3 SMX2
[493] SMX3 BRR2 [494] SMX3 PRP8 [495] SMX3 LSM8
[496] SMX3 SNU66 [497] SNU114 DIB1 [498] PRP8 DIB1
[499] LSM8 DIB1 [500] SNU66 DIB1 [501] DIB1 PRP4
[502] RPN10 RPN1 [503] RPN5 NAS6 [504] RPN12 NAS6
[505] RPN12 RPN1 [506] PRE1 NAS6 [507] RPN8 ATG12
[508] RPN8 NAS6 [509] RPN8 RPN1 [510] ATG12 RPN9
[511] ATG12 NAS6 [512] RPN9 NAS6 [513] RPN9 RPN1
[514] RPN11 NAS6 [515] RPN11 RPN1 [516] NAS6 RPN1
[517] NAS6 RPN6 [518] RPN1 RPN6 [519] SNU114 LSM4
[520] PRP31 LSM4 [521] PRP8 LSM4 [522] PRP3 LSM4
[523] LSM4 PRP4 [524] DHH1 DCP2 [525] DHH1 LSM8
[526] TAF9 TAF14 [527] TAF12 TAF14 [528] TAF1 TAF14
[529] TAF5 TAF14 [530] TAF14 TAF13 [531] TAF14 TAF6
[532] RPN10 PUP1 [533] RPN5 PUP1 [534] PRE1 PUP1
[535] PUP1 SCL1 [536] PUP1 PRE5 [537] IFA38 SHR3
[538] EMP24 AGP1 [539] EMP24 AVT4 [540] EMP24 COS8
[541] EMP24 YPC1 [542] GSF2 SHR3 [543] SEC28 SEC21
[544] SEC28 RET3 [545] SEC28 SEC26 [546] SEC22 BET1

[547] SEC21 RET3 [548] SEC21 SEC26 [549] RET3 SEC26
[550] CEF1 YJU2 [551] SNT309 SNU114 [552] SNT309 PRP45
[553] SNU114 PRP19 [554] SNU114 SYF1 [555] RPF2 IPI3
[556] ARX1 RIX1 [557] ARX1 NOG1 [558] ARX1 IPI3
[559] ARX1 TIF6 [560] RIX1 NOG1 [561] NOG1 IPI3
[562] NSA2 IPI3 [563] RLP7 ARX1 [564] RLP7 RIX1
[565] RLP7 NOG1 [566] ARX1 ERB1 [567] SDA1 ERB1
[568] ERB1 RIX1 [569] ERB1 BUD20 [570] MAK21 NUG1
[571] MAK21 NOG1 [572] HAS1 NUG1 [573] HAS1 NOG1
[574] NUG1 NOG1 [575] NUG1 SSF1 [576] NUG1 TIF6
[577] NOG1 NOP7 [578] ALG1 MST27 [579] MST27 ELO2
[580] TPO3 CAN1 [581] PHO86 SEC63 [582] SEC63 STE24
[583] SEC63 SHR3 [584] STE24 SHR3 [585] PHO86 MST27
[586] BSD2 SPC1 [587] MST27 IFA38 [588] MST27 AGP1
[589] MST27 LAC1 [590] MST27 SPC1 [591] MST27 GAS3
[592] MST27 ELO3 [593] LAC1 PHO88 [594] PHO86 YIL171W
[595] GPI2 YIL171W [596] YHL042W YIL171W [597] CAN1 YIL171W
[598] SPC1 YIL171W [599] ELO3 YIL171W [600] WBP1 AGP1
[601] GPI2 PMP3 [602] MED2 GAL4 [603] MED2 ADA2
[604] GAL11 ADA2 [605] MED8 GAL4 [606] MED8 ADA2
[607] SRB5 ADA2 [608] GAL4 MED7 [609] RLP7 IPI3
[610] YNL132W UTP20 [611] NOP1 UTP20 [612] RPB5 RPC3
[613] RPC3 RPB8 [614] RPN10 HSM3 [615] RPN5 HSM3
[616] RPT4 HSM3 [617] PRE1 HSM3 [618] UBP6 HSM3
[619] HSM3 RAD23 [620] HSM3 RPN11 [621] HSM3 RPT1
[622] RPA12 HHF1 [623] RPA1 HHF1 [624] HHF1 RPB10
[625] RPB10 RPC19 [626] SWD2 CLP1 [627] DBP4 CLP1
[628] CLP1 PAP1 [629] CLP1 PFS2 [630] CLP1 CFT2
[631] CLP1 MPE1 [632] CLP1 YTH1 [633] CLP1 GLC7
[634] CLP1 PTA1 [635] CLP1 REF2 [636] CLP1 PTI1
[637] CLP1 FIP1 [638] SPT5 CKA1 [639] PAF1 CKA1
[640] CDC73 CKA1 [641] LEO1 CKA1 [642] SPT5 RPT1
[643] RTF1 RPT1 [644] CDC73 RPT1 [645] CTR9 UTP22
[646] SPT16 UTP22 [647] TAF12 TAF11 [648] TAF11 TAF6
[649] ADR1 TAF11 [650] ADR1 TAF13 [651] TAF11 ADA2
[652] RPN10 BUD32 [653] RPT6 RPN7 [654] RPN12 BUD32
[655] PRE1 BUD32 [656] YGL004C BUD32 [657] ATG12 BUD32
[658] BUD32 RPN7 [659] BUD32 RPN3 [660] BUD32 RPN11
[661] RPN7 RPN1 [662] RPN3 RPN1 [663] PRP42 SRP1
[664] PHO86 ALG11 [665] AGP1 ALG11 [666] ERG25 ALG11
[667] LAC1 ALG11 [668] SPC1 ALG11 [669] GAS3 ALG11
[670] SAM3 GPI2 [671] SAM3 AVT4 [672] SAM3 ERG25
[673] SAM3 ELO1 [674] ERG11 ALG1 [675] ERG11 YHR140W
[676] ERG11 TSC13 [677] ERG11 IFA38 [678] ERG11 ERG25

[679] ERG11 ELO1 [680] ERG11 GAS3 [681] ERG11 PHO88
[682] MAK21 DBP10 [683] HAS1 NIP7 [684] HAS1 DBP10
[685] ERB1 NIP7 [686] ERB1 DBP10 [687] NIP7 NOP7
[688] NIP7 DBP10 [689] NIP7 TIF6 [690] NIP7 CKA1
[691] RPN10 RPN2 [692] RPT2 RPN2 [693] RPT6 RPN2
[694] RPN12 RPN2 [695] RPN8 RPN2 [696] RPN2 RPT5
[697] RPN2 UBP6 [698] RPN2 RPN9 [699] RPN2 HSM3
[700] RPN2 RPN11 [701] RPN2 RPN3 [702] RPN2 RPN6
[703] RPN2 RPT1 [704] RPF2 YCR072C [705] RLP7 YCR072C
[706] RLP7 NOP7 [707] ERB1 YCR072C [708] ERB1 IPI3
[709] RIX1 NOP7 [710] NOP15 YCR072C [711] YCR072C NOG1
[712] YCR072C NOP7 [713] YCR072C TIF6 [714] NOP7 NSA2
[715] NOP7 IPI3 [716] ENP1 LYS14 [717] YNL132W LYS14
[718] YNL132W UTP6 [719] UTP7 LYS14 [720] LYS14 UTP22
[721] SWC7 ARP4 [722] SWC7 ACT1 [723] SWC7 EPL1
[724] SWR1 EPL1 [725] PRP40 PRP39 [726] SMX2 PRP39
[727] SNU56 PRP39 [728] PRP39 CBC1 [729] PRP39 CBC2
[730] PRP39 PRP42 [731] RPB7 RPC5 [732] RPB9 RPC5
[733] RPC5 RPB1 [734] GCN4 MED7 [735] SRB7 SRB4
[736] SRB7 TAF5 [737] SRB7 SRB2 [738] SRB7 TAF6
[739] NGG1 MED7 [740] SRB4 TAF5 [741] SRB4 TAF6
[742] TAF5 MED7 [743] SRB2 TAF6 [744] NOT2 NOT3
[745] NOT2 CAF130 [746] NOT3 CCR4 [747] NOT5 SSN2
[748] NOT5 POP2 [749] SSN2 NOT4 [750] SSN2 NOT3
[751] SSN2 CAF130 [752] NOT2 POP2 [753] BOS1 SAM1
[754] SAM1 SEC22 [755] SAM1 SEC21 [756] PRE6 PRE4
[757] PRE6 PUP3 [758] PRE4 PRE9 [759] PRE9 PUP3
[760] SMX3 SMD1 [761] PRP31 CUS1 [762] PRP6 CUS1
[763] SMD1 CUS1 [764] SLA1 GCD7 [765] GTS1 ABP1
[766] ABP1 GCD7 [767] RVS167 GCD7 [768] GCD7 YSC84
[769] RSE1 SMD2 [770] RSE1 SMB1 [771] RSE1 SNU71
[772] RSE1 SMD3 [773] SMD2 SMB1 [774] SMD2 CUS1
[775] CBC1 SMB1 [776] SMB1 SNU71 [777] SNU71 CUS1
[778] LUC7 CUS1 [779] CTR9 FKH1 [780] SPT16 FKH1
[781] GPI8 PMT7 [782] PMT7 GPI2 [783] PMT7 GAS3
[784] TSC13 CHO1 [785] IFA38 CHO1 [786] STE24 CHO1
[787] CHO1 ELO1 [788] CHO1 ELO2 [789] HCR1 CKB2
[790] GCN4 GAL4 [791] SRB4 SPT15 [792] CIC1 BRX1
[793] HAS1 BRX1 [794] YTM1 BRX1 [795] NOP7 BRX1
[796] BRX1 SSF1 [797] BRX1 TIF6 [798] BRX1 CKA1
[799] PRE10 PUP3 [800] NUP100 PRE8 [801] KAP95 PRE8
[802] PRE8 NUP2 [803] PRE8 SRP1 [804] PSE1 NUP2
[805] RPT2 RPN7 [806] RPT2 ECM29 [807] RPT6 ECM29
[808] PRE1 ECM29 [809] YGL004C ECM29 [810] RPN8 RPN7

[811] RPN9 RPN7 [812] RPN9 ECM29 [813] RPN7 ECM29
[814] RPN3 ECM29 [815] RPT3 ECM29 [816] AQY1 SHR3
[817] ALG1 SHR3 [818] TPO1 AGP1 [819] PHO86 GAS2
[820] ALG1 GAS2 [821] ALG1 VAP1 [822] GAS2 GPI8
[823] GAS2 IFA38 [824] GAS2 EMP24 [825] GAS2 GSF2
[826] GAS2 ELO2 [827] GAS2 PHO88 [828] GAS2 ELO3
[829] GAS2 YET1 [830] VAP1 GPI8 [831] VAP1 YHR140W
[832] VAP1 GPI2 [833] VAP1 IFA38 [834] VAP1 AGP1
[835] VAP1 ERG25 [836] VAP1 CAN1 [837] VAP1 GAS3
[838] VAP1 PHO88 [839] VAP1 NSG1 [840] AGP1 CAN1
[841] RPF2 URB1 [842] RPF2 MAK5 [843] MAK21 URB1
[844] CIC1 URB1 [845] HAS1 URB1 [846] ERB1 URB1
[847] URB1 YTM1 [848] URB1 NOP7 [849] MAK5 NOP7
[850] SPP381 SMX2 [851] SPP381 SMD1 [852] PRP11 SMD1
[853] RSE1 PRP11 [854] RSE1 PRP21 [855] SPP381 CDC48
[856] SMX2 CDC48 [857] PRP9 CDC48 [858] ROX3 RPB1
[859] MED2 RPB1 [860] RGR1 RPB1 [861] MED7 RPB1
[862] RPT4 RPN1 [863] RPT5 RPN1 [864] UBP6 RPN1
[865] UTP18 SRB4 [866] CKA2 SRB4 [867] SRB4 CKB1
[868] SRB4 CKA1 [869] CIC1 NOG1 [870] YTM1 NOG1
[871] NOP15 MAK5 [872] MAK5 TIF6 [873] MAK5 CKA1
[874] LSM3 LEU2 [875] KEM1 LEU2 [876] LEU2 LSM4
[877] NOG2 RLP7 [878] NOG2 RIX1 [879] NOG2 NOP15
[880] NOG2 NOG1 [881] SMB1 SNU66 [882] ENP1 ASC1
[883] UTP7 CBF5 [884] UTP7 ASC1 [885] PWP2 ASC1
[886] CBF5 ASC1 [887] NOP1 ASC1 [888] HAS1 NOC2
[889] NOP7 NOC2 [890] YTM1 NOC2 [891] NOP15 NOC2
[892] NOC2 TIF6 [893] NOC2 CKA1 [894] AVT4 SPC1
[895] COS8 SPC1 [896] SPC1 YPC1 [897] RPN1 ECM29
[898] RPN5 GFA1 [899] RPT6 GFA1 [900] RPN12 GFA1
[901] PRE1 GFA1 [902] YGL004C GFA1 [903] GFA1 RPN9
[904] GFA1 RPN7 [905] GFA1 RPN11 [906] GFA1 RPN3
[907] GFA1 RPT3 [908] GFA1 RPN6 [909] GFA1 RPT1
[910] WBP1 TSC13 [911] WBP1 YPL264C [912] WBP1 YPC1
[913] PHO86 SWP1 [914] ALG1 SWP1 [915] YHR140W SWP1
[916] GPI8 SWP1 [917] GPI2 SWP1 [918] SUR2 TSC13
[919] SUR2 YPC1 [920] TPO1 YPC1 [921] TSC13 SWP1
[922] YPL264C SWP1 [923] YPL264C YPC1 [924] ERG25 SWP1
[925] GAS3 SWP1 [926] SUR2 AVT4 [927] SUR2 COS8
[928] GSF2 ELO1

3. PPI predictions based on the detected complexes derived from NDComplex (N)

[1] RPN5 RAD23 [2] RPN12 YGL004C [3] RPN12 UBP6
[4] RPN12 ATG12 [5] PRE1 YGL004C [6] PRE1 UBP6

[7] PRE1 ATG12 [8] YGL004C UBP6 [9] YGL004C ATG12
[10] YGL004C RAD23 [11] UBP6 ATG12 [12] UBP6 RAD23
[13] UBP6 RPN7 [14] ATG12 RAD23 [15] RAD23 RPN7
[16] RAD23 RPN11 [17] RPN7 RPN11 [18] LSM3 SMD3
[19] DCP1 PRP24 [20] DCP1 SMD3 [21] DCP1 PAT1
[22] LSM7 SMD3 [23] PRP24 LSM4 [24] PRP24 LSM1
[25] PRP24 SMD3 [26] PRP24 PAT1 [27] LSM1 SMD3
[28] SMD3 PAT1 [29] PHO86 ALG1 [30] PHO86 TPO1
[31] PHO86 IFA38 [32] PHO86 GSF2 [33] ALG1 GSF2
[34] GPI8 TPO1 [35] GPI8 EMP24 [36] GPI8 YPL264C
[37] GPI8 ERG25 [38] GPI2 TPO1 [39] GPI2 TSC13
[40] GPI2 EMP24 [41] TPO1 TSC13 [42] TPO1 EMP24
[43] TPO1 YPL264C [44] IFA38 ELO2 [45] IFA38 PHO88
[46] IFA38 ELO3 [47] IFA38 YET1 [48] EMP24 YPL264C
[49] YPL264C GAS3 [50] GSF2 ELO2 [51] GSF2 PHO88
[52] GSF2 YET1 [53] ELO2 PHO88 [54] ELO2 ELO3
[55] ELO2 YET1 [56] PHO88 ELO3 [57] PHO88 YET1
[58] ELO3 YET1 [59] SUI1 SUA7 [60] TIF34 HCR1
[61] SUA7 HCR1 [62] HCR1 NIP1 [63] NOT2 CAF16
[64] SSN3 CAF40 [65] SSN3 CAF16 [66] CAF40 CAF16
[67] CAF16 NOT1 [68] PHO86 STE24 [69] PHO86 ELO1
[70] ALG1 STE24 [71] ALG1 AVT4 [72] ALG1 ELO1
[73] ALG1 YPC1 [74] BSD2 YHR140W [75] BSD2 GPI8
[76] BSD2 AVT4 [77] BSD2 ERG25 [78] BSD2 COS8
[79] GPI8 AVT4 [80] GPI8 COS8 [81] TSC13 YPC1
[82] IFA38 STE24 [83] STE24 ELO2 [84] STE24 PHO88
[85] STE24 ELO3 [86] STE24 YET1 [87] AVT4 COS8
[88] AVT4 GAS3 [89] ERG25 COS8 [90] ERG25 ELO1
[91] COS8 GAS3 [92] ELO1 YPC1 [93] GAS3 YPC1
[94] BOS1 GPT2 [95] SEC22 GPT2 [96] SEC21 GPT2
[97] RET1 GPT2 [98] MUD1 SMD2 [99] MUD1 LUC7
[100] PRP40 SNU56 [101] PRP40 SMD2 [102] PRP40 CBC2
[103] PRP40 PRP42 [104] SMX2 NAM8 [105] SMX2 CBC2
[106] SMX2 PRP42 [107] SNU56 SMD2 [108] SNU56 CBC1
[109] SNU56 PRP42 [110] SMD2 SNP1 [111] SMD2 PRP42
[112] YHC1 CBC2 [113] CBC2 PRP42 [114] LSM3 DCP2
[115] KEM1 DCP1 [116] KEM1 LSM6 [117] KEM1 LSM7
[118] KEM1 PRP24 [119] KEM1 LSM1 [120] KEM1 LSM5
[121] DCP2 LSM6 [122] DCP2 LSM7 [123] DCP2 PRP24
[124] DCP2 LSM5 [125] DCP2 SMD3 [126] GCN4 SPT7
[127] GCN4 TAF10 [128] TAF9 SPT15 [129] SPT7 TAF1
[130] SPT7 SPT15 [131] NGG1 TAF10 [132] SPT15 TAF10
[133] YHR140W AGP1 [134] TSC13 AGP1 [135] TSC13 SHR3
[136] AGP1 AVT4 [137] AGP1 COS8 [138] AGP1 YPC1

[139] ELO2 SHR3 [140] SHR3 PHO88 [141] SHR3 ELO3
[142] SHR3 YET1 [143] BSD2 EMP24 [144] BSD2 YPL264C
[145] BSD2 CAN1 [146] GPI8 CAN1 [147] GPI2 NSG1
[148] TSC13 CAN1 [149] EMP24 CAN1 [150] EMP24 NSG1
[151] YPL264C CAN1 [152] YPL264C NSG1 [153] ERG25 CAN1
[154] ERG25 NSG1 [155] CAN1 GAS3 [156] GCN4 TAF12
[157] GCN4 TAF13 [158] TAF9 TAF12 [159] TAF12 ADA2
[160] SPT15 TAF13 [161] TAF13 ADA2 [162] WBP1 YHR140W
[163] WBP1 GPI8 [164] WBP1 ERG25 [165] WBP1 GAS3
[166] ALG1 SUR2 [167] SUR2 ERG25 [168] SUR2 GAS3
[169] PHO86 CSG2 [170] ALG1 CSG2 [171] GPI8 CSG2
[172] CSG2 COS8 [173] COS8 CAN1 [174] TAF5 GCN5
[175] TAF5 UBP8 [176] UBP8 TAF10 [177] UBP8 TAF6
[178] UBP8 ADA2 [179] ALG1 AGP1 [180] ALG1 LAC1
[181] BSD2 AGP1 [182] YHR140W LAC1 [183] GPI8 AGP1
[184] AGP1 YPL264C [185] GSF2 LAC1 [186] LAC1 ELO2
[187] LAC1 NSG1 [188] NSG1 YET1 [189] PHO86 BAP3
[190] GPI2 BAP3 [191] SMX2 BRR2 [192] SMX2 PRP8
[193] SMX2 LSM8 [194] SNU114 PRP6 [195] SNU114 SMB1
[196] SNU114 PRP3 [197] PRP6 SMB1 [198] PRP8 SMD1
[199] LSM8 SMD1 [200] SMB1 PRP3 [201] SMB1 PRP4
[202] PRP3 SMD1 [203] SMD1 PRP4 [204] RSE1 PRP43
[205] RSE1 SNU114 [206] PRP8 PRP45 [207] SPP381 PRP8
[208] SPP381 LSM8 [209] SPP381 SMD3 [210] SNU114 SNU66
[211] SNU114 SNU23 [212] PRP31 SNU23 [213] PRP6 SNU66
[214] PRP6 SNU23 [215] PRP8 SNU23 [216] LSM8 SNU23
[217] PRP3 SNU66 [218] PRP3 SNU23 [219] SNU66 SNU23
[220] RPN10 PPH22 [221] RPN5 PRE4 [222] RPN5 PPH22
[223] RPN5 PUP3 [224] RPN5 PRE8 [225] PRE4 PUP3
[226] PRE4 PRE5 [227] PUP3 PRE5 [228] LSM3 EDC3
[229] EDC3 LSM4 [230] EDC3 LSM1 [231] EDC3 PAT1
[232] RRP43 RRP6 [233] RRP43 SKI7 [234] RRP43 RRP42
[235] RRP6 SKI7 [236] RRP6 RRP4 [237] RRP6 RRP42
[238] SKI7 RRP42 [239] SKI7 SRP1 [240] RRP42 SRP1
[241] YNL132W NOP1 [242] UTP7 SOF1 [243] KRR1 UTP22
[244] SOF1 UTP22 [245] TIF5 CKB2 [246] SUI1 CKB2
[247] TIF34 CKB2 [248] SUA7 CKB2 [249] CTF8 POL30
[250] CTF18 POL30 [251] LSM3 SMX2 [252] LSM3 PRP6
[253] PHO86 AQY1 [254] PHO86 TPO3 [255] ALG1 AQY1
[256] GPI8 TPO3 [257] AQY1 GPI2 [258] AQY1 TSC13
[259] AQY1 IFA38 [260] AQY1 GAS3 [261] GPI2 TPO3
[262] TSC13 TPO3 [263] ERG25 TPO3 [264] PHO86 SLY41
[265] YHR140W SLY41 [266] ELO1 SLY41 [267] ELO2 SLY41
[268] BOS1 DSL1 [269] SEC27 DSL1 [270] SEC22 DSL1

[271] DSL1 SEC21 [272] SAM3 YHR140W [273] SAM3 TSC13
[274] SAM3 GAS3 [275] ALG1 GIT1 [276] GIT1 GPI8
[277] GIT1 YHR140W [278] GIT1 TSC13 [279] GIT1 IFA38
[280] GIT1 GSF2 [281] GIT1 ELO2 [282] GIT1 GAS3
[283] GIT1 NSG1 [284] GIT1 FET3 [285] TSC13 FET3
[286] GAS3 FET3 [287] NSG1 FET3 [288] TAF9 SPT20
[289] TAF1 SPT20 [290] TAF5 SPT20 [291] SPT20 TAF6
[292] GCN4 ADR1 [293] TAF9 ADR1 [294] NGG1 ADR1
[295] RNA14 SWD2 [296] RNA14 YTH1 [297] RNA14 GLC7
[298] RNA14 PTI1 [299] SWD2 DBP4 [300] SWD2 PCF11
[301] SWD2 PAP1 [302] SWD2 YTH1 [303] SWD2 PTI1
[304] SWD2 FIP1 [305] CFT1 DBP4 [306] DBP4 PCF11
[307] DBP4 MPE1 [308] DBP4 YTH1 [309] PCF11 PFS2
[310] PCF11 YTH1 [311] PCF11 GLC7 [312] PCF11 REF2
[313] PCF11 PTI1 [314] PCF11 FIP1 [315] PFS2 GLC7
[316] GLC7 PTI1 [317] YSH1 PTI1 [318] REF2 FIP1
[319] GIN4 HSP26 [320] BNI5 SHS1 [321] BNI5 HSP26
[322] CDC3 HSP26 [323] SPT7 ADR1 [324] SIN4 SRB2
[325] ROX3 SRB6 [326] ROX3 MED2 [327] RGR1 SRB2
[328] DCP2 RPS23A [329] DCP2 RPS28B [330] EDC3 RPS23A
[331] RPS23A RPS28B [332] RRP43 MTR3 [333] RRP6 MTR3
[334] SKI7 MTR3 [335] RRP4 MTR3 [336] MTR3 SRP1
[337] RPN10 RPT5 [338] RPT2 RPT4 [339] RPT2 RPT6
[340] RPT2 RPN8 [341] RPT2 RPT5 [342] RPT2 RPN9
[343] RPT2 RPN3 [344] RPT4 PRE1 [345] RPT4 RPN8
[346] RPT4 RPN9 [347] RPT4 RAD23 [348] RPT4 RPN3
[349] RPT4 RPN11 [350] RPT6 RPN8 [351] RPT6 RPT5
[352] RPT6 RPN9 [353] RPT6 RPN3 [354] RPN8 RPT5
[355] RPN8 RPN3 [356] RPT5 UBP6 [357] RPT5 RPN9
[358] RPT5 RPN3 [359] RPT5 RPN11 [360] RPN9 RPN3
[361] SSN2 GAL11 [362] SRB6 SFL1 [363] MED2 SFL1
[364] SFL1 GAL11 [365] SFL1 MED7 [366] VID21 HTZ1
[367] VID21 ARP4 [368] VID21 ACT1 [369] VID21 TRA1
[370] VID21 RVB2 [371] SWC4 SAP185 [372] HTZ1 ESA1
[373] HTZ1 SAP185 [374] ACT1 TRA1 [375] TRA1 RVB2
[376] ESA1 RVB2 [377] SAP185 RVB2 [378] SAP185 YAF9
[379] RVB2 EPL1 [380] RPB7 IKI3 [381] RPB5 TFG1
[382] RPB9 IKI3 [383] RPB2 TFG1 [384] TFG1 RPB4
[385] TFG1 IKI3 [386] TFG1 RPB1 [387] RPB3 RPB1
[388] RPN10 PRE10 [389] RPN10 POC4 [390] PRE6 PRE10
[391] PRE6 PRE9 [392] PRE6 PRE5 [393] PRE10 PRE9
[394] PRE10 PRE8 [395] PRE1 POC4 [396] PPH22 POC4
[397] PRE9 PRE5 [398] POC4 PRE2 [399] SPP381 LSM5
[400] BRR2 LSM5 [401] PRP31 LSM5 [402] PRP8 LSM5

[403] PRP3 LSM5 [404] SNU23 LSM5 [405] YHR140W YHL042W
[406] YHR140W SPC1 [407] YHL042W ELO1 [408] GPI8 SPC1
[409] TSC13 SPC1 [410] AGP1 SPC1 [411] YPL264C SPC1
[412] SPC1 GAS3 [413] RPB5 RPC17 [414] RPB5 RPC2
[415] RPC17 RPC2 [416] RPC2 RPB8 [417] RPB2 RPB8
[418] RPB4 RPB8 [419] RPB8 IKI3 [420] SMB1 DIB1
[421] DIB1 SMD1 [422] PHO86 PFA4 [423] ALG1 PFA4
[424] GPI2 FET3 [425] SUR2 FET3 [426] ERG25 FET3
[427] ELO2 PFA4 [428] PFA4 PHO88 [429] PFA4 YET1
[430] SWD2 SHG1 [431] SWD2 SDC1 [432] SET3 CPR1
[433] ZDS1 CPR1 [434] ZDS1 HST1 [435] ARC18 MYO5
[436] ARC15 MYO5 [437] ARC35 MYO5 [438] CDC28 ORC5
[439] CDC28 ORC4 [440] CDC28 ORC3 [441] RRP43 RRP40
[442] RRP6 RRP40 [443] RRP4 RRP40 [444] RRP40 CSL4
[445] RRP40 SRP1 [446] MUD1 SRP1 [447] PRP40 SRP1
[448] SMX2 SRP1 [449] YHC1 SRP1 [450] SNU71 SRP1
[451] LUC7 SRP1 [452] CDC27 APC9 [453] APC4 CDC16
[454] APC1 APC9 [455] APC9 CDC26 [456] APC9 DOC1
[457] DNL4 ERB1 [458] DNL4 GLC7 [459] DNL4 NOP6
[460] RPF2 GLC7 [461] CIC1 NOP7 [462] CIC1 TIF6
[463] NOP2 NOP15 [464] NOP2 GLC7 [465] NOP15 GLC7
[466] NOP15 NOP6 [467] GLC7 NOP6 [468] GLC7 TIF6
[469] SIN4 SRB8 [470] RGR1 SRB8 [471] SRB8 SRB2
[472] MAK21 NOP6 [473] MAK21 TIF6 [474] MAK21 CKA1
[475] RPF2 NOP4 [476] RPF2 CKA1 [477] CIC1 HAS1
[478] HAS1 YTM1 [479] YTM1 SSF1 [480] YTM1 CKA1
[481] NOP15 SSF1 [482] NOP15 CKA1 [483] NOP4 NOP6
[484] NOP4 TIF6 [485] NOP6 SSF1 [486] NOP6 CKA1
[487] OST3 PKC1 [488] OST4 PKC1 [489] OST5 PKC1
[490] PKC1 OST2 [491] STT3 SWP1 [492] SMX3 SMX2
[493] SMX3 BRR2 [494] SMX3 PRP8 [495] SMX3 LSM8
[496] SMX3 SNU66 [497] SNU114 DIB1 [498] PRP8 DIB1
[499] LSM8 DIB1 [500] SNU66 DIB1 [501] DIB1 PRP4
[502] RPN10 RPN1 [503] RPN5 NAS6 [504] RPN12 NAS6
[505] RPN12 RPN1 [506] PRE1 NAS6 [507] RPN8 ATG12
[508] RPN8 NAS6 [509] RPN8 RPN1 [510] ATG12 RPN9
[511] ATG12 NAS6 [512] RPN9 NAS6 [513] RPN9 RPN1
[514] RPN11 NAS6 [515] RPN11 RPN1 [516] NAS6 RPN1
[517] NAS6 RPN6 [518] RPN1 RPN6 [519] SNU114 LSM4
[520] PRP31 LSM4 [521] PRP8 LSM4 [522] PRP3 LSM4
[523] LSM4 PRP4 [524] DHH1 DCP2 [525] DHH1 LSM8
[526] TAF9 TAF14 [527] TAF12 TAF14 [528] TAF1 TAF14
[529] TAF5 TAF14 [530] TAF14 TAF13 [531] TAF14 TAF6
[532] RPN10 PUP1 [533] RPN5 PUP1 [534] PRE1 PUP1

[535] PUP1 SCL1 [536] PUP1 PRE5 [537] IFA38 SHR3
[538] EMP24 AGP1 [539] EMP24 AVT4 [540] EMP24 COS8
[541] EMP24 YPC1 [542] GSF2 SHR3 [543] SEC28 SEC21
[544] SEC28 RET3 [545] SEC28 SEC26 [546] SEC22 BET1
[547] SEC21 RET3 [548] SEC21 SEC26 [549] RET3 SEC26
[550] CEF1 YJU2 [551] SNT309 SNU114 [552] SNT309 PRP45
[553] SNU114 PRP19 [554] SNU114 SYF1 [555] RPF2 IPI3
[556] ARX1 RIX1 [557] ARX1 NOG1 [558] ARX1 IPI3
[559] ARX1 TIF6 [560] RIX1 NOG1 [561] NOG1 IPI3
[562] NSA2 IPI3 [563] RLP7 ARX1 [564] RLP7 RIX1
[565] RLP7 NOG1 [566] ARX1 ERB1 [567] SDA1 ERB1
[568] ERB1 RIX1 [569] ERB1 BUD20 [570] MAK21 NUG1
[571] MAK21 NOG1 [572] HAS1 NUG1 [573] HAS1 NOG1
[574] NUG1 NOG1 [575] NUG1 SSF1 [576] NUG1 TIF6
[577] NOG1 NOP7 [578] ALG1 MST27 [579] MST27 ELO2
[580] TPO3 CAN1 [581] PHO86 SEC63 [582] SEC63 STE24
[583] SEC63 SHR3 [584] STE24 SHR3 [585] PHO86 MST27
[586] BSD2 SPC1 [587] MST27 IFA38 [588] MST27 AGP1
[589] MST27 LAC1 [590] MST27 SPC1 [591] MST27 GAS3
[592] MST27 ELO3 [593] LAC1 PHO88 [594] PHO86 YIL171W
[595] GPI2 YIL171W [596] YHL042W YIL171W [597] CAN1 YIL171W
[598] SPC1 YIL171W [599] ELO3 YIL171W [600] WBP1 AGP1
[601] GPI2 PMP3 [602] MED2 GAL4 [603] MED2 ADA2
[604] GAL11 ADA2 [605] MED8 GAL4 [606] MED8 ADA2
[607] SRB5 ADA2 [608] GAL4 MED7 [609] RLP7 IPI3
[610] YNL132W UTP20 [611] NOP1 UTP20 [612] RPB5 RPC3
[613] RPC3 RPB8 [614] RPN10 HSM3 [615] RPN5 HSM3
[616] RPT4 HSM3 [617] PRE1 HSM3 [618] UBP6 HSM3
[619] HSM3 RAD23 [620] HSM3 RPN11 [621] HSM3 RPT1
[622] RPA12 HHF1 [623] RPA1 HHF1 [624] HHF1 RPB10
[625] RPB10 RPC19 [626] SWD2 CLP1 [627] DBP4 CLP1
[628] CLP1 PAP1 [629] CLP1 PFS2 [630] CLP1 CFT2
[631] CLP1 MPE1 [632] CLP1 YTH1 [633] CLP1 GLC7
[634] CLP1 PTA1 [635] CLP1 REF2 [636] CLP1 PTI1
[637] CLP1 FIP1 [638] SPT5 CKA1 [639] PAF1 CKA1
[640] CDC73 CKA1 [641] LEO1 CKA1 [642] SPT5 RPT1
[643] RTF1 RPT1 [644] CDC73 RPT1 [645] CTR9 UTP22
[646] SPT16 UTP22 [647] TAF12 TAF11 [648] TAF11 TAF6
[649] ADR1 TAF11 [650] ADR1 TAF13 [651] TAF11 ADA2
[652] RPN10 BUD32 [653] RPT6 RPN7 [654] RPN12 BUD32
[655] PRE1 BUD32 [656] YGL004C BUD32 [657] ATG12 BUD32
[658] BUD32 RPN7 [659] BUD32 RPN3 [660] BUD32 RPN11
[661] RPN7 RPN1 [662] RPN3 RPN1 [663] PRP42 SRP1
[664] PHO86 ALG11 [665] AGP1 ALG11 [666] ERG25 ALG11

[667] LAC1 ALG11 [668] SPC1 ALG11 [669] GAS3 ALG11
[670] SAM3 GPI2 [671] SAM3 AVT4 [672] SAM3 ERG25
[673] SAM3 ELO1 [674] ERG11 ALG1 [675] ERG11 YHR140W
[676] ERG11 TSC13 [677] ERG11 IFA38 [678] ERG11 ERG25
[679] ERG11 ELO1 [680] ERG11 GAS3 [681] ERG11 PHO88
[682] MAK21 DBP10 [683] HAS1 NIP7 [684] HAS1 DBP10
[685] ERB1 NIP7 [686] ERB1 DBP10 [687] NIP7 NOP7
[688] NIP7 DBP10 [689] NIP7 TIF6 [690] NIP7 CKA1
[691] RPN10 RPN2 [692] RPT2 RPN2 [693] RPT6 RPN2
[694] RPN12 RPN2 [695] RPN8 RPN2 [696] RPN2 RPT5
[697] RPN2 UBP6 [698] RPN2 RPN9 [699] RPN2 HSM3
[700] RPN2 RPN11 [701] RPN2 RPN3 [702] RPN2 RPN6
[703] RPN2 RPT1 [704] RPF2 YCR072C [705] RLP7 YCR072C
[706] RLP7 NOP7 [707] ERB1 YCR072C [708] ERB1 IPI3
[709] RIX1 NOP7 [710] NOP15 YCR072C [711] YCR072C NOG1
[712] YCR072C NOP7 [713] YCR072C TIF6 [714] NOP7 NSA2
[715] NOP7 IPI3 [716] ENP1 LYS14 [717] YNL132W LYS14
[718] YNL132W UTP6 [719] UTP7 LYS14 [720] LYS14 UTP22
[721] SWC7 ARP4 [722] SWC7 ACT1 [723] SWC7 EPL1
[724] SWR1 EPL1 [725] PRP40 PRP39 [726] SMX2 PRP39
[727] SNU56 PRP39 [728] PRP39 CBC1 [729] PRP39 CBC2
[730] PRP39 PRP42 [731] RPB7 RPC5 [732] RPB9 RPC5
[733] RPC5 RPB1 [734] GCN4 MED7 [735] SRB7 SRB4
[736] SRB7 TAF5 [737] SRB7 SRB2 [738] SRB7 TAF6
[739] NGG1 MED7 [740] SRB4 TAF5 [741] SRB4 TAF6
[742] TAF5 MED7 [743] SRB2 TAF6 [744] NOT2 NOT3
[745] NOT2 CAF130 [746] NOT3 CCR4 [747] NOT5 SSN2
[748] NOT5 POP2 [749] SSN2 NOT4 [750] SSN2 NOT3
[751] SSN2 CAF130 [752] NOT2 POP2 [753] BOS1 SAM1
[754] SAM1 SEC22 [755] SAM1 SEC21 [756] PRE6 PRE4
[757] PRE6 PUP3 [758] PRE4 PRE9 [759] PRE9 PUP3
[760] SMX3 SMD1 [761] PRP31 CUS1 [762] PRP6 CUS1
[763] SMD1 CUS1 [764] SLA1 GCD7 [765] GTS1 ABP1
[766] ABP1 GCD7 [767] RVS167 GCD7 [768] GCD7 YSC84
[769] RSE1 SMD2 [770] RSE1 SMB1 [771] RSE1 SNU71
[772] RSE1 SMD3 [773] SMD2 SMB1 [774] SMD2 CUS1
[775] CBC1 SMB1 [776] SMB1 SNU71 [777] SNU71 CUS1
[778] LUC7 CUS1 [779] CTR9 FKH1 [780] SPT16 FKH1
[781] GPI8 PMT7 [782] PMT7 GPI2 [783] PMT7 GAS3
[784] TSC13 CHO1 [785] IFA38 CHO1 [786] STE24 CHO1
[787] CHO1 ELO1 [788] CHO1 ELO2 [789] HCR1 CKB2
[790] GCN4 GAL4 [791] SRB4 SPT15 [792] CIC1 BRX1
[793] HAS1 BRX1 [794] YTM1 BRX1 [795] NOP7 BRX1
[796] BRX1 SSF1 [797] BRX1 TIF6 [798] BRX1 CKA1

[799] PRE10 PUP3 [800] NUP100 PRE8 [801] KAP95 PRE8
[802] PRE8 NUP2 [803] PRE8 SRP1 [804] PSE1 NUP2
[805] RPT2 RPN7 [806] RPT2 ECM29 [807] RPT6 ECM29
[808] PRE1 ECM29 [809] YGL004C ECM29 [810] RPN8 RPN7
[811] RPN9 RPN7 [812] RPN9 ECM29 [813] RPN7 ECM29
[814] RPN3 ECM29 [815] RPT3 ECM29 [816] AQY1 SHR3
[817] ALG1 SHR3 [818] TPO1 AGP1 [819] PHO86 GAS2
[820] ALG1 GAS2 [821] ALG1 VAP1 [822] GAS2 GPI8
[823] GAS2 IFA38 [824] GAS2 EMP24 [825] GAS2 GSF2
[826] GAS2 ELO2 [827] GAS2 PHO88 [828] GAS2 ELO3
[829] GAS2 YET1 [830] VAP1 GPI8 [831] VAP1 YHR140W
[832] VAP1 GPI2 [833] VAP1 IFA38 [834] VAP1 AGP1
[835] VAP1 ERG25 [836] VAP1 CAN1 [837] VAP1 GAS3
[838] VAP1 PHO88 [839] VAP1 NSG1 [840] AGP1 CAN1
[841] RPF2 URB1 [842] RPF2 MAK5 [843] MAK21 URB1
[844] CIC1 URB1 [845] HAS1 URB1 [846] ERB1 URB1
[847] URB1 YTM1 [848] URB1 NOP7 [849] MAK5 NOP7
[850] SPP381 SMX2 [851] SPP381 SMD1 [852] PRP11 SMD1
[853] RSE1 PRP11 [854] RSE1 PRP21 [855] SPP381 CDC48
[856] SMX2 CDC48 [857] PRP9 CDC48 [858] ROX3 RPB1
[859] MED2 RPB1 [860] RGR1 RPB1 [861] MED7 RPB1
[862] RPT4 RPN1 [863] RPT5 RPN1 [864] UBP6 RPN1
[865] UTP18 SRB4 [866] CKA2 SRB4 [867] SRB4 CKB1
[868] SRB4 CKA1 [869] CIC1 NOG1 [870] YTM1 NOG1
[871] NOP15 MAK5 [872] MAK5 TIF6 [873] MAK5 CKA1
[874] LSM3 LEU2 [875] KEM1 LEU2 [876] LEU2 LSM4
[877] NOG2 RLP7 [878] NOG2 RIX1 [879] NOG2 NOP15
[880] NOG2 NOG1 [881] SMB1 SNU66 [882] ENP1 ASC1
[883] UTP7 CBF5 [884] UTP7 ASC1 [885] PWP2 ASC1
[886] CBF5 ASC1 [887] NOP1 ASC1 [888] HAS1 NOC2
[889] NOP7 NOC2 [890] YTM1 NOC2 [891] NOP15 NOC2
[892] NOC2 TIF6 [893] NOC2 CKA1 [894] AVT4 SPC1
[895] COS8 SPC1 [896] SPC1 YPC1 [897] RPN1 ECM29
[898] RPN5 GFA1 [899] RPT6 GFA1 [900] RPN12 GFA1
[901] PRE1 GFA1 [902] YGL004C GFA1 [903] GFA1 RPN9
[904] GFA1 RPN7 [905] GFA1 RPN11 [906] GFA1 RPN3
[907] GFA1 RPT3 [908] GFA1 RPN6 [909] GFA1 RPT1
[910] WBP1 TSC13 [911] WBP1 YPL264C [912] WBP1 YPC1
[913] PHO86 SWP1 [914] ALG1 SWP1 [915] YHR140W SWP1
[916] GPI8 SWP1 [917] GPI2 SWP1 [918] SUR2 TSC13
[919] SUR2 YPC1 [920] TPO1 YPC1 [921] TSC13 SWP1
[922] YPL264C SWP1 [923] YPL264C YPC1 [924] ERG25 SWP1
[925] GAS3 SWP1 [926] SUR2 AVT4 [927] SUR2 COS8
[928] GSF2 ELO1

S4. PPIs predicted at least twice based on the prediction set N (N^+)

[1] RPN5 RAD23 [2] RPN12 YGL004C [3] RPN12 UBP6
[4] RPN12 ATG12 [5] PRE1 YGL004C [6] PRE1 UBP6
[7] PRE1 ATG12 [8] YGL004C UBP6 [9] YGL004C ATG12
[10] YGL004C RAD23 [11] UBP6 RAD23 [12] UBP6 RPN7
[13] RAD23 RPN7 [14] RAD23 RPN11 [15] RPN7 RPN11
[16] LSM3 SMD3 [17] DCP1 PRP24 [18] DCP1 SMD3
[19] DCP1 PAT1 [20] LSM7 SMD3 [21] PRP24 LSM4
[22] PRP24 LSM1 [23] PRP24 SMD3 [24] PRP24 PAT1
[25] LSM1 SMD3 [26] SMD3 PAT1 [27] PHO86 ALG1
[28] PHO86 TPO1 [29] PHO86 IFA38 [30] PHO86 GSF2
[31] ALG1 GSF2 [32] GPI8 TPO1 [33] GPI8 EMP24
[34] GPI8 YPL264C [35] GPI8 ERG25 [36] GPI2 TPO1
[37] GPI2 TSC13 [38] GPI2 EMP24 [39] TPO1 TSC13
[40] TPO1 YPL264C [41] IFA38 ELO2 [42] IFA38 PHO88
[43] IFA38 ELO3 [44] IFA38 YET1 [45] EMP24 YPL264C
[46] YPL264C GAS3 [47] GSF2 ELO2 [48] GSF2 PHO88
[49] GSF2 YET1 [50] ELO2 PHO88 [51] ELO2 ELO3
[52] ELO2 YET1 [53] PHO88 ELO3 [54] PHO88 YET1
[55] ELO3 YET1 [56] SUI1 SUA7 [57] TIF34 HCR1
[58] SUA7 HCR1 [59] HCR1 NIP1 [60] PHO86 STE24
[61] PHO86 ELO1 [62] ALG1 STE24 [63] ALG1 AVT4
[64] ALG1 ELO1 [65] ALG1 YPC1 [66] BSD2 YHR140W
[67] BSD2 GPI8 [68] BSD2 AVT4 [69] BSD2 ERG25
[70] BSD2 COS8 [71] GPI8 AVT4 [72] GPI8 COS8
[73] TSC13 YPC1 [74] IFA38 STE24 [75] STE24 ELO2
[76] STE24 PHO88 [77] STE24 YET1 [78] AVT4 COS8
[79] AVT4 GAS3 [80] ERG25 COS8 [81] ERG25 ELO1
[82] COS8 GAS3 [83] ELO1 YPC1 [84] GAS3 YPC1
[85] MUD1 SMD2 [86] MUD1 LUC7 [87] PRP40 SNU56
[88] PRP40 SMD2 [89] PRP40 CBC2 [90] PRP40 PRP42
[91] SMX2 NAM8 [92] SMX2 CBC2 [93] SMX2 PRP42
[94] SNU56 SMD2 [95] SNU56 CBC1 [96] SNU56 PRP42
[97] SMD2 SNP1 [98] SMD2 PRP42 [99] YHC1 CBC2
[100] CBC2 PRP42 [101] LSM3 DCP2 [102] KEM1 DCP1
[103] KEM1 LSM6 [104] KEM1 LSM7 [105] KEM1 PRP24
[106] KEM1 LSM1 [107] KEM1 LSM5 [108] DCP2 LSM6
[109] DCP2 LSM7 [110] DCP2 PRP24 [111] DCP2 LSM5
[112] GCN4 SPT7 [113] GCN4 TAF10 [114] TAF9 SPT15
[115] SPT7 TAF1 [116] SPT7 SPT15 [117] NGG1 TAF10
[118] SPT15 TAF10 [119] YHR140W AGP1 [120] TSC13 AGP1
[121] TSC13 SHR3 [122] AGP1 AVT4 [123] AGP1 COS8
[124] AGP1 YPC1 [125] ELO2 SHR3 [126] SHR3 PHO88
[127] SHR3 ELO3 [128] SHR3 YET1 [129] BSD2 YPL264C

[130] GPI8 CAN1 [131] GPI2 NSG1 [132] TSC13 CAN1
[133] EMP24 CAN1 [134] EMP24 NSG1 [135] YPL264C NSG1
[136] ERG25 CAN1 [137] ERG25 NSG1 [138] CAN1 GAS3
[139] GCN4 TAF12 [140] GCN4 TAF13 [141] TAF9 TAF12
[142] TAF12 ADA2 [143] SPT15 TAF13 [144] TAF13 ADA2
[145] WBP1 YHR140W [146] WBP1 GPI8 [147] WBP1 ERG25
[148] WBP1 GAS3 [149] ALG1 SUR2 [150] SUR2 ERG25
[151] SUR2 GAS3 [152] GPI8 CSG2 [153] CSG2 COS8
[154] COS8 CAN1 [155] ALG1 AGP1 [156] BSD2 AGP1
[157] YHR140W LAC1 [158] GPI8 AGP1 [159] AGP1 YPL264C
[160] GSF2 LAC1 [161] NSG1 YET1 [162] SMX2 BRR2
[163] SMX2 PRP8 [164] SMX2 LSM8 [165] SNU114 PRP6
[166] SNU114 SMB1 [167] SNU114 PRP3 [168] PRP6 SMB1
[169] PRP8 SMD1 [170] LSM8 SMD1 [171] SMB1 PRP3
[172] SMB1 PRP4 [173] PRP3 SMD1 [174] SMD1 PRP4
[175] RSE1 PRP43 [176] RSE1 SNU114 [177] PRP8 PRP45
[178] SPP381 PRP8 [179] SPP381 LSM8 [180] SPP381 SMD3
[181] SNU114 SNU66 [182] SNU114 SNU23 [183] PRP31 SNU23
[184] PRP6 SNU66 [185] PRP6 SNU23 [186] PRP8 SNU23
[187] LSM8 SNU23 [188] PRP3 SNU66 [189] PRP3 SNU23
[190] RPN10 PPH22 [191] RPN5 PPH22 [192] RPN5 PUP3
[193] RPN5 PRE8 [194] PRE4 PUP3 [195] PRE4 PRE5
[196] PUP3 PRE5 [197] EDC3 LSM4 [198] EDC3 LSM1
[199] RRP43 RRP6 [200] RRP43 SKI7 [201] RRP43 RRP42
[202] RRP6 SKI7 [203] RRP6 RRP4 [204] RRP6 RRP42
[205] SKI7 RRP42 [206] SKI7 SRP1 [207] RRP42 SRP1
[208] YNL132W NOP1 [209] UTP7 SOF1 [210] KRR1 UTP22
[211] SOF1 UTP22 [212] TIF5 CKB2 [213] SUI1 CKB2
[214] TIF34 CKB2 [215] SUA7 CKB2 [216] PHO86 AQY1
[217] PHO86 TPO3 [218] GPI2 TPO3 [219] SAM3 YHR140W
[220] SAM3 TSC13 [221] SAM3 GAS3 [222] GCN4 ADR1
[223] TAF9 ADR1 [224] NGG1 ADR1 [225] RNA14 SWD2
[226] RNA14 YTH1 [227] RNA14 GLC7 [228] RNA14 PTI1
[229] SWD2 DBP4 [230] SWD2 PCF11 [231] SWD2 PAP1
[232] SWD2 YTH1 [233] SWD2 PTI1 [234] SWD2 FIP1
[235] CFT1 DBP4 [236] DBP4 PCF11 [237] DBP4 MPE1
[238] DBP4 YTH1 [239] PCF11 PFS2 [240] PCF11 YTH1
[241] PCF11 GLC7 [242] PCF11 REF2 [243] PCF11 PTI1
[244] PCF11 FIP1 [245] PFS2 GLC7 [246] GLC7 PTI1
[247] YSH1 PTI1 [248] REF2 FIP1 [249] GIN4 HSP26
[250] BNI5 SHS1 [251] BNI5 HSP26 [252] CDC3 HSP26
[253] SIN4 SRB2 [254] ROX3 SRB6 [255] ROX3 MED2
[256] RGR1 SRB2 [257] RRP43 MTR3 [258] RRP6 MTR3
[259] SKI7 MTR3 [260] RRP4 MTR3 [261] MTR3 SRP1

[262] RPN10 RPT5 [263] RPT2 RPT4 [264] RPT2 RPT6
[265] RPT2 RPN8 [266] RPT2 RPT5 [267] RPT2 RPN9
[268] RPT2 RPN3 [269] RPT4 PRE1 [270] RPT4 RPN8
[271] RPT4 RPN9 [272] RPT4 RAD23 [273] RPT4 RPN3
[274] RPT4 RPN11 [275] RPT6 RPN8 [276] RPT6 RPT5
[277] RPT6 RPN9 [278] RPT6 RPN3 [279] RPN8 RPT5
[280] RPN8 RPN3 [281] RPT5 UBP6 [282] RPT5 RPN9
[283] RPT5 RPN3 [284] RPT5 RPN11 [285] RPN9 RPN3
[286] SWC4 SAP185 [287] HTZ1 ESA1 [288] HTZ1 SAP185
[289] ACT1 TRA1 [290] SAP185 YAF9 [291] RVB2 EPL1
[292] RPB7 IKI3 [293] RPB9 IKI3 [294] RPB3 RPB1
[295] RPN10 PRE10 [296] PRE6 PRE9 [297] PRE6 PRE5
[298] PRE10 PRE9 [299] PRE10 PRE8 [300] PRE9 PRE5
[301] YHR140W YHL042W [302] YHR140W SPC1 [303] AGP1 SPC1
[304] SPC1 GAS3 [305] RPB5 RPC17 [306] RPB5 RPC2
[307] RPC17 RPC2 [308] RPC2 RPB8 [309] RPB2 RPB8
[310] MUD1 SRP1 [311] YHC1 SRP1 [312] SNU71 SRP1
[313] LUC7 SRP1 [314] CIC1 NOP7 [315] CIC1 TIF6
[316] NOP2 NOP15 [317] NOP15 NOP6 [318] MAK21 NOP6
[319] MAK21 TIF6 [320] MAK21 CKA1 [321] RPF2 NOP4
[322] RPF2 CKA1 [323] CIC1 HAS1 [324] HAS1 YTM1
[325] YTM1 SSF1 [326] YTM1 CKA1 [327] NOP15 SSF1
[328] NOP15 CKA1 [329] NOP4 NOP6 [330] NOP4 TIF6
[331] NOP6 SSF1 [332] NOP6 CKA1 [333] SMX3 SMX2
[334] SMX3 BRR2 [335] RPN10 RPN1 [336] RPN12 RPN1
[337] RPN8 RPN1 [338] ATG12 RPN9 [339] RPN9 RPN1
[340] RPN11 RPN1 [341] RPN1 RPN6 [342] EMP24 AGP1
[343] SEC28 SEC21 [344] SEC28 RET3 [345] SEC28 SEC26
[346] SEC22 BET1 [347] SEC21 RET3 [348] SEC21 SEC26
[349] RET3 SEC26 [350] RPF2 IPI3 [351] ARX1 RIX1
[352] ARX1 NOG1 [353] ARX1 TIF6 [354] RIX1 NOG1
[355] NOG1 IPI3 [356] NSA2 IPI3 [357] RLP7 ARX1
[358] RLP7 RIX1 [359] RLP7 NOG1 [360] SDA1 ERB1
[361] ERB1 RIX1 [362] ERB1 BUD20 [363] MAK21 NOG1
[364] HAS1 NOG1 [365] NOG1 NOP7 [366] STE24 SHR3
[367] WBP1 AGP1 [368] RLP7 IPI3 [369] YNL132W UTP20
[370] RPN10 HSM3 [371] RPN5 HSM3 [372] RPT4 HSM3
[373] PRE1 HSM3 [374] UBP6 HSM3 [375] HSM3 RAD23
[376] HSM3 RPN11 [377] HSM3 RPT1 [378] TAF11 TAF6
[379] RPN10 BUD32 [380] RPT6 RPN7 [381] RPN12 BUD32
[382] PRE1 BUD32 [383] YGL004C BUD32 [384] ATG12 BUD32
[385] BUD32 RPN7 [386] BUD32 RPN3 [387] BUD32 RPN11
[388] RPN7 RPN1 [389] RPN3 RPN1 [390] SWC7 ARP4
[391] SWC7 ACT1 [392] SWC7 EPL1 [393] SWR1 EPL1

[394] PRP40 PRP39 [395] SMX2 PRP39 [396] SNU56 PRP39
[397] PRP39 CBC1 [398] PRP39 PRP42 [399] SRB4 TAF6
[400] SRB2 TAF6 [401] NOT2 NOT3 [402] NOT2 CAF130
[403] NOT3 CCR4 [404] NOT5 SSN2 [405] NOT5 POP2
[406] SSN2 NOT4 [407] SSN2 CAF130 [408] NOT2 POP2
[409] PRE6 PRE4 [410] PRE6 PUP3 [411] PRE4 PRE9
[412] PRE9 PUP3 [413] SMD1 CUS1 [414] RPT2 RPN7
[415] RPT2 ECM29 [416] RPT6 ECM29 [417] PRE1 ECM29
[418] YGL004C ECM29 [419] RPN8 RPN7 [420] RPN9 RPN7
[421] RPN9 ECM29 [422] RPN7 ECM29 [423] RPN3 ECM29
[424] RPT3 ECM29 [425] RPF2 MAK5 [426] MAK5 NOP7
[427] SPP381 SMX2 [428] UBP6 RPN1 [429] CIC1 NOG1
[430] YTM1 NOG1 [431] NOP15 MAK5 [432] MAK5 TIF6
[433] MAK5 CKA1 [434] HAS1 NOC2 [435] NOP7 NOC2
[436] SUR2 TSC13 [437] SUR2 YPC1
